# A Review of High-Entropy Alloys (HEAs): Phase Stability, Nanostructures, Mechanical Behavior, and Future Engineering Applications

**DOI:** 10.3390/nano16211378

**Published:** 2026-10-28

**Authors:** Benedict Raphael Soesanto, Lee Zhi Szen, Jason Chua Teng Sern, Lee Jing Xian, Ha Shou Yi, Sachin Sharma Ashok Kumar

**Affiliations:** 1 School of Engineering, Taylor’s University, 1 Jalan Taylor’s, Subang Jaya 47500, Selangor, Malaysia; 2 Clean Technology Impact Lab, Taylor’s University, Subang Jaya 47500, Selangor, Malaysia

**Keywords:** high-entropy alloys (HEAs), nanostructures, phase formations, high-entropy effect

## Abstract

High-entropy alloys (HEAs) are revolutionary materials containing five or more primary elements in near-equiatomic amounts to produce an alloy with high configurational entropy that stabilizes at a simple solid solution. Unlike conventional alloys, which are based on one dominant element, HEAs represent a novel advanced material that has shown great properties. These properties are the reason for scientific interest, as they have potential industrial applications due to their exceptional mechanical, thermal, and chemical properties. This paper aims to explore the different rules and principles that dictate their phase formation, including the thermodynamic and kinetic factors that are involved in the development of their phases and nanostructures. The four key effects associated with HEAs will also be explored: the high-entropy effect, severe lattice distortion effect, sluggish diffusion effect, and cocktail effect. The relationship between their unique phase formations and nanostructures and their properties will be discussed, with a view to optimizing them to prioritize certain properties for targeted applications. The mechanisms that occur and dictate these effects on the HEA for its strengthening and deformation will be analyzed to identify the benefits and trade-offs associated with modifying alloy composition and processing parameters. The fabrication methods for different types of HEAs will be reviewed along with current and emerging industrial applications of HEAs, especially in fields that require superior mechanical performance and environmental resistance. Thus, this review highlights their behavior, optimization, and potential applications as the next generation of materials.

## 1. Introduction

High-entropy alloys (or HEAs) are characterized as alloys that consist of five or more main elements. The composition of each component of the alloy ranges between 5% and 35%. HEAs consist of multiple principal elements in equimolar or near-equimolar ratios, where they tend to form single-phase solutions [1,2,3,4]. Due to the composition and the complexity of their microstructure, HEAs possess the following characteristics: high mixing entropy, resulting in low Gibbs free energy, causing HEAs to form stable, solid forms and simple, stable, solid solutions [5,6,7,8] with face-centered-cubic (FCC), body-centered-cubic (BCC) and/or, hexagonal closed-pack (HCP) structures rather than intermetallic phases comprising traditional alloys [9]; severe lattice distortion, which results in high strength induced by the solid solution strengthening effect [10,11]; sluggish diffusion [12,13], which increases the thermal stability of HEAs [14]; and the cocktail effect [15], whereby adjusting the composition of HEAs would enable the manipulation of properties [16]. The characteristics of HEAs include high strength [17], excellent corrosion resistance [18,19,20,21], good wear resistance [22], and unique electrical and magnetic properties [23]. Moreover, studies have indicated that HEAs’ high-temperature stability and corrosion resistance enable them to be promising candidates for demanding applications, including use as catalysts [24] and in high-temperature water vapor conditions such as those observed in thermal power plants [25], ocean-based oil detection environments [26], extreme irradiation environments [27], and cryogenic conditions [28]. Citing their excellent mechanical properties in both high [29] and low temperatures [30], and good fatigue resistance [31,32,33,34,35,36,37], HEAs remain a promising candidate for use as structural materials in harsh and corrosive environments [16]. 

Early research conducted by Yeh et al. proposed that the formation of HEAs is single-phase only, with the single phases including BCC, FCC, and HCP. However, in recent years, research has indicated that the composition of HEAs can be manipulated to give rise to the formation of multiple phases and even intermetallic precipitates, which can result in improvements in certain properties. The onset of specific phases or mixtures of phases has its own unique advantages and drawbacks. For example, HEAs of single BCC phases exhibit high hardness and strength characteristics, but possess poor ductility and a high susceptibility to corrosion. Single FCC-structured HEAs show excellent plasticity but poor strength. HEAs with HCP phases are uncommon and possess high work hardening and ductility. Moreover, dual-phase HEAs exhibit a collection of good properties. Research conducted by Fang et al. [38] reported that a CoCrFeMnNi HEA with a dual-phase microstructure of 16.7% FCC and 83.3% HCP phases exhibited superior strength and plasticity compared to single-phase FCC-based or HCP-based HEAs. They attributed this enhancement to the strengthening derived from the FCC/HCP interfaces. Another study examining Al_x_CoCrCuFeNi HEA with BCC/FCC dual phases noticed that the solid solution strengthening of both phases was unequal—the BCC phase was measured at 1286 MPa, while the FCC phase was 379 MPa due to higher lattice distortion and modulus mismatch in BCC due to the presence of aluminum in the alloy. However, the HEA of HCP + FCC possesses a higher hardness than any single-phase HEA. This is a result of solid solution strengthening from BCC coupled with interfacial hardening of FCC/BCC [39]. The FeCoCrNiMn HEA, consisting of FCC/BCC dual phases, exhibited excellent strain-hardening capabilities and high tensile plasticity; such enhanced mechanical properties can be attributed to strain-induced transformation from the FCC to the BCC phase [40].

Studies have indicated that the formation of phases in HEAs can be predicted before production. One of several prediction methods involves computing the valence electron concentration (VEC) of the alloy. The VEC indicates that the FCC structure is formed only when the VEC is greater than eight. However, when the VEC ranges between 6.87 < VEC < 8, the HEA structure will comprise a dual-phase FCC + BCC [41]. When the VEC < 6.87, the HEA will only form a BCC structure [42]. This prediction parameter, however, includes some limitations since it does not take into account the effect of temperature or non-equiatomic compositions. From the prediction workflows, customization and manipulation of the HEA structure (to suit the desired application) are made possible through incorporating certain elements. For example, addition of chromium and aluminum stabilizes the BCC phase, whereas elements like cobalt and nickel aid the formation of FCC [43]. 

Another predictive method used to determine the phase of HEA includes the use of the Law of Thermodynamics relating to Gibbs free energy. When analyzing the factors that influence HEA phases, kinetic actions may be ignored in favor of Gibbs free energy. Gibbs free energy can be represented as a property of enthalpy and entropy, as shown in the equation below [44]:

Δ*G_mix_* = Δ*H_mix_* − *T*Δ*S_mix_* (1)

where

ΔG_mix_ = Gibbs free energy;

ΔH_mix_ = the mixing enthalpy;

ΔS_mix =_ the mixing entropy.

The equation indicates that both the enthalpy and entropy of mixing dictate the phases of HEA. When atomic size differences are very small, the ∆*H_mix_* is not sufficiently negative; hence, the atoms can easily substitute for each other to form disordered solid solutions (such as FCC, BCC, or HCP single-phase HEAs) because the energy is not low enough to form intermetallic compounds. On the other hand, when atomic differences are sufficiently large to prevent substitution, the ∆*H_mix_* is very negative, where ordered intermetallic compounds form instead. However, if the size difference is excessively large, amorphous structures form instead of crystalline HEA; high temperatures coupled with high mixing entropy make the TΔS_mix_ very negative, which causes a negative ΔG and stabilizes disordered solid solutions, even when ΔH_mix_ is moderately negative—this explains why HEAs favor simple single-phase structures over complex intermetallics that would normally form in multi-component systems.

Another prediction method used to determine solid solution formation in HEAs is known as the Hume–Rothery rule, which states that when the difference in the atomic size of the alloy exceeds 15%, the formation of solid solution is not possible. The use of the solid solution factor Ω can be used to predict whether the alloy will form a solid solution or an intermetallic compound using the following mathematical equation:

(2) $$Ω=T_{m}\frac{∆S_{\mathrm{mix}}}{\left|∆H_{\mathrm{mix}}\right|}$$

where

ΔH_mix_ = the mixing enthalpy;

ΔS_mix =_ the mixing entropy.

Here, Tm is denoted as the melting temperature of the alloy in Kelvin (K), ∆*S_mix_* denotes the mixing entropy, and ∆*H_mix_* denotes the mixing enthalpy of the alloy. Hence, if the solid solution factor Ω exceeds the value of 1, the formation of solution is possible. Additionally, when ∆*H_mix_* ranges from −15 kJ/mol to 5 kJ/mol, the formation of a single-phase solid-solution alloy is possible [6].

Despite substantial research and numerous documented research articles on HEAs, existing knowledge remains fragmented across distinct topics, including phase stability, strengthening mechanisms, processing routes, individual HEA classes, and specific application areas [45]. In particular, the relationship between alloy composition, phase prediction, fabrication conditions, resulting microstructure, and engineering performance remains scattered despite their strong interdependence. Although there have been numerous empirical parameters proposed to predict HEA phase stability, their applicability is limited to alloy systems where overlapping phase regions can reduce predictive reliability. Therefore, these empirical criteria should be critically compared rather than evaluated individually [46]. Empirical phase-selection parameters exhibit individual limitations, while identical or similar HEA compositions can develop different phase constitutions and properties that are dependent on the fabrication routes employed [47]. Hence, there remains a need for an integrated assessment that relates fundamental HEA design principles to process-induced microstructural evolution and application performance. The present review addresses these research gaps by performing a comprehensive examination of phase-stability criteria and their limitations, strengthening and deformation mechanisms, fabrication-dependent phase evolution, and the properties and applications of major HEA classes, including FCC-based, refractory, nanostructured, eutectic, and high-entropy superalloy systems. Using such an approach, this review interconnects composition, processing, microstructure, properties, and applications in a collective manner, while highlighting the key scientific and practical challenges that limit the wider engineering use of HEAs.

## 2. Methodology

The literature reviewed in this study was identified through searches of Scopus, Web of Science, or ScienceDirect using keywords related to high-entropy alloys (HEAs), including “high-entropy alloys,” “phase stability,” “fabrication methods,” “microstructure,” “mechanical properties,” “alloy classification,” and “engineering applications.” Boolean operators were used to combine relevant terms where appropriate. The search covered publications from 2000 to 2026, based on the actual search period.

The literature was screened according to its relevance to the scope of the present review. Publications were included when they addressed one or more of the following areas: (i) fundamental concepts and compositional design of HEAs; (ii) thermodynamic and kinetic factors governing phase formation and stability; (iii) strengthening mechanisms and mechanical behavior; (iv) fabrication and processing techniques; (v) different classes and types of HEAs; (vi) nanostructured and nanocrystalline HEAs where relevant to the review scope; and (vii) current and potential engineering applications. Studies that were unrelated to HEAs, did not contribute substantially to the topics covered in this review, or were outside the defined scope were excluded.

For the final synthesis, the selected studies were compared qualitatively to identify relationships between core HEA concepts → phase stability → strengthening/mechanical behavior → fabrication → types/classes of HEAs → applications. The literature was synthesized to highlight how compositional design and thermodynamic considerations influence phase constitution and how fabrication conditions subsequently affect the resulting microstructure and properties. This review also compares the characteristics and potential applications of different HEA classes, including conventional HEAs, refractory HEAs, eutectic HEAs, high-entropy superalloys, and nanostructured HEAs. This approach provides an integrated overview of the development of HEAs from their fundamental principles and phase stability through processing, material classification, properties, and potential engineering applications.

## 3. Phase Formation and Stability

Phase formation is one of the most important aspects when determining the structure of a high-entropy alloy (HEA). Unlike conventional alloys, HEAs typically form simple crystal structures, namely face-centered cubic (FCC), body-centered cubic (BCC), hexagonal close-packed (HCP), and intermetallic compounds (IM) [48]. These differences correspond to different properties such as strength, ductility, and thermal stability.

### 3.1. Configurational Entropy

In a paper by Nataraj [49], HEAs can be modeled as an ideal solution, thus leading to the configurational entropy equation to be applied to find its configurational entropy of mixing (Δ*S_mix_*):

(3) $$\Delta S_{\mathrm{mix}}=-R\sum_{i=1}^{n}c_{i}\ln c_{i}$$

where

R = the universal gas constant;

*c_i_* = the molar fraction of the *i^th^* element;

n = the total number of components.

From this equation, it can generally be confirmed that HEAs do have higher entropy, as their results lie in the middle of the triangle. It is also indicated that there can be many different compositions of HEAs, as there are many different principal alloys found within HEAs [49].

The large number of elemental compositions increases the likelihood of solid-state solutions forming. Additionally, many other phases can also form, from intermetallic compounds to metallic glasses [50,51]. For HEAs, this has a configurational entropy of 1.5R, which is around 12 J/mol K [51].

### 3.2. Atomic Size Difference

To further add to the parameters, an additional atomic size difference could be added, as proposed by Wu and team [42], using the modified Kadomtsev–Petviashvili equation by Zhang and Lv [52]. The equation is as follows [44]:

(4) $$\delta \%=100\%\times \sqrt{\sum_{i=1}^{n}c_{i}{\left(1-\frac{r_{i}}{\sum_{j=1}^{n}c_{j}r_{j}}\right)}^{2}}$$

where

n = the number of different elements;

*c_j_* = the elemental composition of the *i^th^* and *j^th^* elements; 

*r_i_*,*r_j_* = the atomic radius of the *i^th^* and *j^th^* elements.

A study by Wu and team [42] concluded that a larger atomic size difference correlates to a higher activation energy needed for grain growth as well as a higher degree of lattice distortion, causing atoms to move toward their most stable state. When reducing the nuclear size difference, the single-phase solution will be enhanced, and potentially, an amorphous structure will be formed [44].

### 3.3. Mixing Enthalpy

Similarly to the previous section, Ye and team [1] used the modified Kadomtsev–Petviashvili equation by Zhang and Lv [52] to form a new mixing equation for HEAs. The equation is as follows:

(5) $$\Delta H_{\mathrm{mix}}=\sum_{i=1,i\neq j}^{n}4{\Delta H}_{\mathrm{ij}}^{\mathrm{mix}}c_{i}c_{j}$$

where

${\Delta H}_{\mathrm{ij}}^{\mathrm{mix}}$ = the binary alloy mixing enthalpy;

*c_j_* = the elemental composition of the *i^th^* and *j^th^* elements. 

The mixing enthalpy, along with the atomic size difference, is a great indicator of the different phases formed. A single-phase solid solution is generally found within −15 kJ/mol < Δ*H_mix_* < 5 kJ/mol and 0 < *δ* < 5. It can also be seen that when Δ*H_mix_* decreases and the atomic size difference increases, it becomes a multiphase solid solution, and when it goes further, it may become an amorphous solid solution or even a metallic glass [53].

### 3.4. Combining Effect

Yang [54] proposed a new parameter that uses the melting temperature of the alloy, where phase formation generally occurs; the equation can be seen below:

(6) $$T_{m}=\sum_{i=1}^{n}c_{i}{\left(T_{m}\right)}_{i}$$

where

*T_m_* = is the average melting temperature.

The value of Ω was used to find the ratio between the three factors. When Ω = 1, the mixture is at its critical value to form a solid solution. Thus, when Ω > 1, the value of *T_m_*Δ*S_mix_* is higher than Δ*H_mix_*, which indicates that a solid solution is being formed, resulting in the HEA being composed mostly of solid solution. When Ω < 1, on the other hand, intermetallic compounds and segregation are formed prior to the solid solution, leading to a greater multiphase solution [54].

### 3.5. Critical Solution Temperature

The critical solution temperature is the temperature below the alloy’s melting point and just below it when the combining effect parameter starts to take place [44]. During cooling, the alloy reaches this temperature first before undergoing solidification and phase formation. Thus, this parameter must be considered when analyzing the phase formation and microstructural development of HEAs during the cooling process. The equation can be seen as follows:

(7) $$T_{C}=\frac{\left|{\Delta H}_{\mathrm{mix}}\right|}{{\Delta S}_{\mathrm{mix}}}$$

where

ΔH_mix_ = the mixing enthalpy;

ΔS_mix =_ the mixing entropy.

### 3.6. Packing Density Factor

Packing density also plays a role in the HEA’s mixing entropy, which is said to have a higher effect when temperatures are lower. The relationship is expressed by an equation presented in Mansoori’s [55] and Ye’s paper [1], where the following equation was proposed:

(8) $$S_{T}(c_{i},r_{i},\xi )={S_{C}\left(c_{i}\right)+S}_{E}(c_{i},r_{i},\xi )$$

where

*S_T_* = the total configurational entropy of mixing per sphere;

*S_C_* = the configurational entropy of mixing for an ideal solution;

*S_E_* = excess entropy of mixing;

*c_i_* = the elemental composition of the i^th^ elements;

*r_i_* = the atomic radius of the i^th^ elements;

*ξ* = the packing density factor.

As seen in the equation, the total configurational entropy and excess entropy are functions of the packing density factor. The excess entropy of mixing is generally negative and influenced by the packing density factor (*ξ*). When *ξ* is small, the excess entropy of mixing also decreases. Thus, the total configurational entropy of mixing per sphere is at its maximum when the packing density factor is at its smallest, where temperatures are generally higher. Therefore, its packing density has a major influence on the total configurational entropy of mixing at low temperatures [1].

### 3.7. Valence Electron Concentration

Valence electron concentration (VEC) is used to predict the phase of a HEA from FCC, BCC, HCP, and intermetallic compounds. The Hume–Rothery rule dictates that the VEC is a critical parameter in dictating the crystallinity of a solid solution when there is no strong atomic size effect [56]. Balaji concluded that HEAs with an FCC phase have a VEC > 8.0, whereas those with a BCC have a VEC of <6.87, with a combination of the two or intermetallic phases in between the two values [57].

(9) $${\mathrm{VEC}}_{\mathrm{avg}}=\sum_{i=1}^{n}c_{i}{\left(\mathrm{VEC}\right)}_{i}$$

where

(*VEC*)*_i_* = the valence electron concentration of the i^th^ elements;

*c_i_* = the elemental composition of the i^th^ elements. 

From the relationship between the VEC and atomic size difference, it was indicated that the Al–Ti–Cu–Zn-based HEA was more likely to form an FCC crystal structure, whereas an Al–Co–Cr–Fe-based HEA is more likely to form a BCC structure [44].

### 3.8. Single-Phase Solution Parameter

Ye and team [58] also proposed a new parameter to take account of the configuration of HEAs at high and low packing densities. The equation is as follows:

(10) $$\phi =\frac{S_{c}-|\Delta H_{\mathrm{mix}}|/T_{m}}{\left|S_{E}\right|}$$

where

***S_C_*** = the configurational entropy of mixing for an ideal solution;

***S_E_*** = the excess entropy of mixing; 

ΔH_mix_ = the mixing enthalpy;

*T_m_* = the average melting temperature.

When *ϕ* has a larger value, the HEA is most likely to form a single-phase solid solution. The HEA had a critical *ϕ* of 20, with those larger having a single-phase solution and those with a lower value being multiphase or of an amorphous structure [1].

### 3.9. Parameter Limitations

These parameters are great in identifying which phase a given solution forms. However, some limitations still exist, as depicted in Table 1 [50,51,53,57,58,59].

## 4. Core Effects

There are four major core effects, each with its own characteristics and advantages: the high-entropy effect, severe lattice distortion effect (SLD-effect), sluggish diffusion effect, and cocktail effect. A thorough understanding of these four key effects is critical for the principled and exact design of HEAs. 

### 4.1. High-Entropy Effect

**Figure 1:**
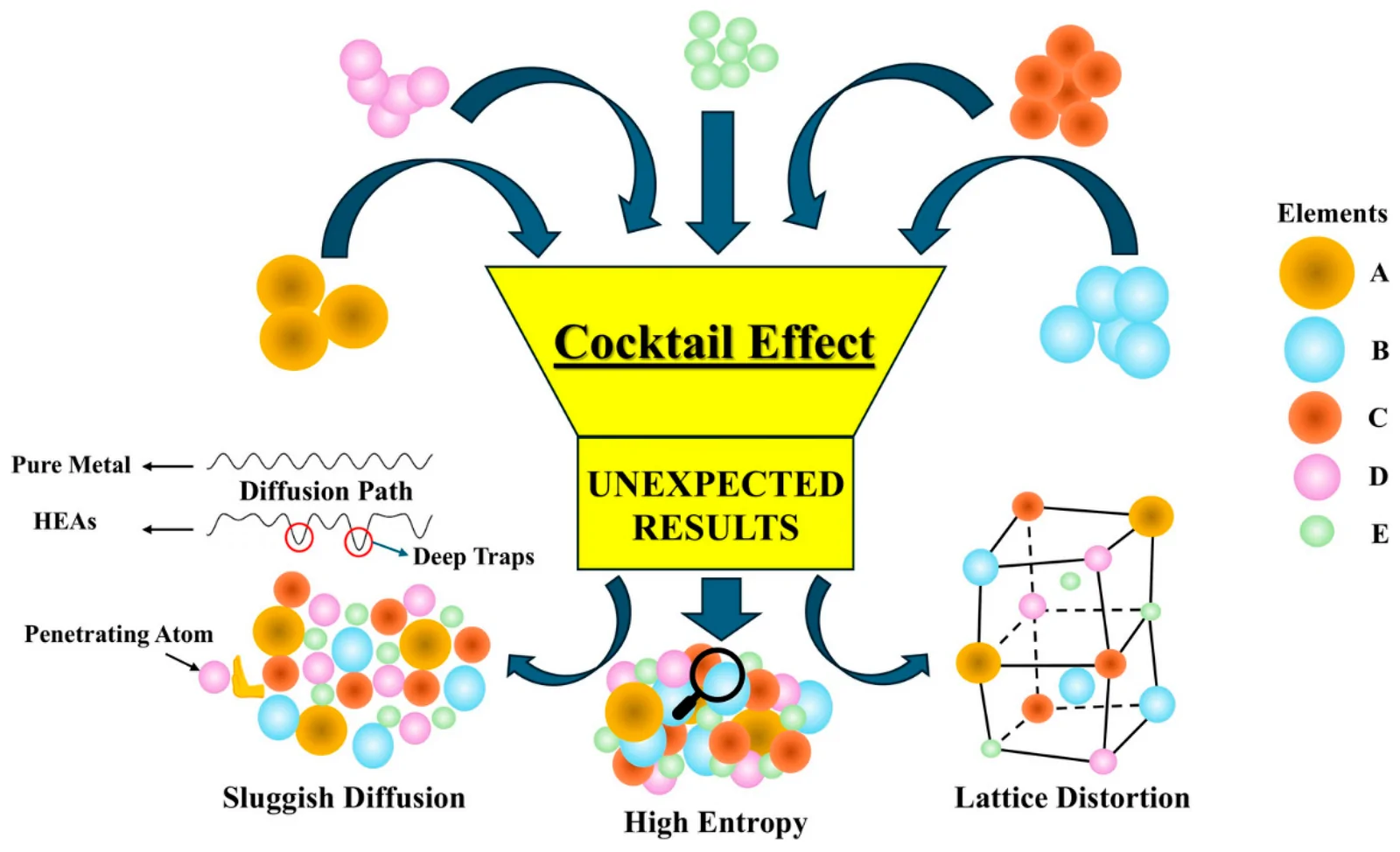
Schematic representation of the four core effects in HEAs. Reprinted from Ref. [46].

The high-entropy effect, initially proposed by Yeh et al., refers to the phenomenon where alloys that contain five or more elements will exhibit a high degree of mixing entropy and have a higher possibility of forming single-phase solid solutions than intermetallic compounds [60]. Despite the high-entropy effect’s elemental and chemical complexity, it still tends to form solid solutions with FCC or BCC simple structures. Thus, the high-entropy effect is one of the most prominent characteristics of HEAs. The disordered distribution of atoms that constitute HEAs is shown in Figure 1 [46]. Furthermore, the entropy effects of HEAs are inversely related to Gibbs free energy. The greater the entropy at higher temperatures, the lower the Gibbs free energy, which favors the formation and stability of the alloy. Aside from this, it helps to reduce the tendency to separate while creating complex structures [46]. However, this does not imply that a simple solid solution can form at a higher temperature [15].

### 4.2. Severe Lattice Distortion Effect (SLD Effect)

**Figure 2:**
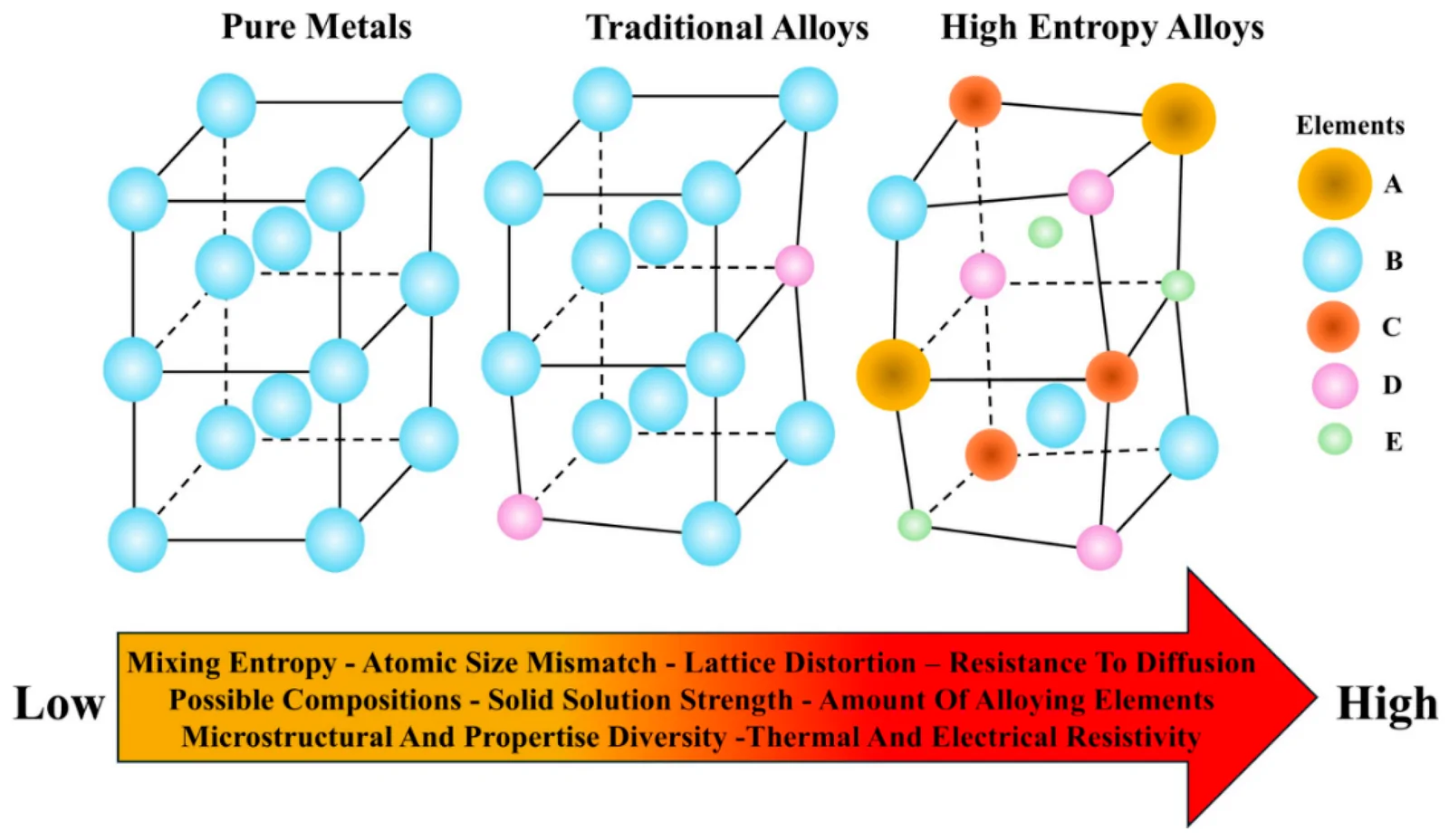
Schematic diagram of a hypothetical material with a BCC structure in a pure metal, traditional alloy, and high-entropy alloy with A–E atoms. Reprinted from Ref. [46].

The severe lattice distortion effect is well known for the deviation or displacement of the constituent atoms in an alloy from their ideal lattice positions due to the introduction of substantial solute atoms into a solvent matrix due to their differences in atomic size, mass, and bonding characteristics, which result in severe distortions in the lattice structure, as shown in Figure 2 [46,61]. Larger metallic atoms compress the surrounding atoms, whereas smaller metallic atoms form a strain field and cause a change in the bulk lattice parameter [62]. The localized lattice distortions surrounding the solute atoms will interact with moving dislocations, thereby hindering dislocation motion, where it requires a greater applied stress for deformation to occur, resulting in solid solution strengthening [63]. Additionally, according to a study by Balaji V. and Anthony Xavior M., the BCC structure can exhibit more pronounced distortion effects than the FCC structure due to its differences in atomic arrangement, deformation behavior, and the existence of more active slip planes [44]. As a result, these characteristics have made the BCC structure more susceptible to distortion and deformation. Therefore, a number of studies and research have suggested that severe lattice dislocation contributes significantly to the exceptional properties of HEA, including its high yield strength, improved wear resistance, and enhanced thermal stability [64]. 

### 4.3. Sluggish Diffusion Effect

The sluggish diffusion effect often refers to a reduction in the atomic diffusion rate caused by the highly complex atomic arrangement present in the multi-principal alloy, which is based on a kinetic nature. The concept was initially proposed during the early development of HEAs by J.W. Yeh et al., based on observations of slow precipitation, grain growth, and phase transformation kinetics [3]. As shown previously in Figure 2, the atoms may encounter more irregular diffusion pathways in HEAs compared with pure metals [46]. Furthermore, atoms tend to diffuse more slowly compared to traditional alloys due to the high and complicated structural disorder caused by the presence of numerous elements with various atomic sizes and complex atomic interactions in HEAs, which require more energy to move [46]. These unique properties were able to increase stability at high temperatures and resistance to creep formation, and increase the activation energy, based on a research paper published by Pickering EJ and Jones NG [62]. 

Tsai et al. studied and published the first direct diffusion research, which used the diffusion couple approach [13]. Initially, the authors used a quasi-binary technique to explore the diffusion coefficient of the Co-Cr-Fe Mn-Ni system, imposing gradients of only two elements in each diffusion couple, and comparing it to the diffusion coefficients of its pure metallic elements [65]. From the studies, the authors reported that the Co-Cr-Fe Mn-Ni HEA has a lower diffusion coefficient compared to its reference pure metallic elements. Additionally, the activation energy was found to be relatively higher than that of its constituent elements. As a result, a hypothesis was proposed, with the findings explaining why the sluggish diffusion effect was found in HEAs. However, since only a few of the high-entropy systems were experimentally studied over the years, there was still uncertainty, and the diffusion behavior of HEAs remains under debate regarding the hypothesis being made [66]. Subsequent studies have questioned whether these hypotheses can be generalized for all HEA systems [65]. For instance, Juliusz Dąbrowa et al. suggested that the observed diffusion behavior may be strongly influenced by the specific alloy composition, particularly the presence of manganese content in the HEA system, rather than solely by the high configurational entropy of the alloy [65]. A more recent review by V. Verma et al. (2024) has also concluded that there remains insufficient experimental evidence to establish sluggish diffusion as a general characteristic of HEAs, partly because reliable measurements of diffusion in quaternary and high-order systems remain challenging [66]. Therefore, this suggests that sluggish diffusion should not be regarded as a universal characteristic of all HEA systems. 

### 4.4. Cocktail Effect

Chen et al. was the first to present the cocktail effect by utilizing an alloy design and its characteristics [67]. Cocktail effects in HEAs are often defined as a synergistic interaction between various elements or atoms that contribute to increasing the material’s characteristics beyond the average of its constituent elements. Unlike conventional alloys that were previously or presently used, where only one base element dominates the composition, HEAs consist of several elements that are mixed in near-equiatomic ratios, creating a “multimetallic cocktail” that results in unpredictable and diverse properties due to complexity [68]. 

Similarly to how the final flavor of a cocktail changes from the simple sum of its ingredients, the properties exhibited by HEAs cannot be predicted solely from the rule of mixtures. Table 2 below presents the structural effects of HEAs when adjustments in composition or the addition of various other elements were made in several studies. Therefore, this shows that the performance and phase transformation of HEAs were directly dependent on the type of metallic elements being added into the HEA structure and the adjustments made to the composition. 

## 5. Strengthening and Deformation Mechanism

### 5.1. Solid Solution Strengthening

#### 5.1.1. Fundamental Mechanism of Solid Solution Strengthening

Solid solution strengthening (SSS) is one of the primary strengthening mechanisms observed in HEAs, especially in single-phase solid solutions of HEAs. This strengthening mechanism occurs due to the interaction between the dislocation distribution and different constituents of the atoms within the crystal lattice of HEAs. Unlike conventional alloys where a minute amount of solute atoms is added into a dominant solvent, HEAs generally contain several principal elements in relatively high concentrations. Henceforth, the distinction between solute and solvent is more ambiguous and less clear, where each constituent element has the potential to contribute to the strengthening of the alloy. The random distribution of the different atoms creates a fluctuation of intermolecular and intramolecular forces within the local lattice environment, which increases the resistance of forces among the dislocated atoms. Therefore, a greater amount of stress is required to initiate plastic deformation, hence leading to an incremental yield strength and hardness of the HEA, which explains the strengthening mechanism [74,75]. 

#### 5.1.2. Atomic Size Mismatch and Crystal Lattice Distortion Effect

One of the main factors contributing to the SSS is the difference in atomic sizes among the varying constituent elements within the HEA. When atoms possessing different atomic radii occupy a common crystal lattice site, the surrounding atoms will experience displacement and dislocation from their ideal lattice position. Under the effect of dislocation of atoms, larger atoms will compress the surrounding lattice, while smaller atoms generate a local tensile strain field, creating a localized lattice distortion throughout the HEA. As previously established, due to the presence of large amounts of varying principal elements within a HEA, this distortion of the lattice structure is particularly significant, causing the interaction of the dislocation effect with the local strain fields generated by the constituent atoms of the principal elements. Henceforth, additional stress needs to be applied to overcome the localized obstacles caused by the atomic mismatched distortion effect, thereby increasing the strength of HEAs [74,76]. 

#### 5.1.3. Shear Modulus Mismatch and Dislocation-Atom Interaction Energy

Aside from differences in atomic sizes that lead to the distortion effect, the difference in the shear modulus between the principal elements also contributes significantly to the SSS. The shear modulus represents the resistance of a material against shear deformation, where differences in shear modulus between the varying principal elements within a HEA could generate a variation in the local elastic response of the lattice structure. Consequently, when a dislocation passes through regions within the structure with atoms possessing distinct elastic properties, the dislocation experiences unique interaction energy with each distinct elastic property. This fluctuation of dislocation-atom interaction energy restricts the movement of the dislocation effect and increases the stress required for plastic deformation, hence indicating the increase in strength within the HEA [75]. 

### 5.2. Grain Boundary Strengthening

#### 5.2.1. Fundamental Mechanism of Grain Boundary Strengthening in HEA

Grain boundary strengthening (GBS) is an important strengthening mechanism in HEAs. The grain boundaries act as barriers to traversing dislocation motion, therefore increasing the resistance of the HEA material towards plastic deformation. In HEAs, neighboring grains possess different crystallographic orientations; hence, the slip systems activated within them are generally unaligned adjacent grains within the structure of the HEA. When dislocations traveling through a grain approach a grain boundary, the continued propagation of dislocations requires either dislocation transmission into the neighboring grain or the activation of another compatible slip system. This incompatibility increases the stress required for further plastic deformation, resulting in an increase in the yield strength of the HEA [77,78].

The strengthening contribution from grain boundaries is significantly dependent on the grain size. As the average grain size decreases, the number of grain boundaries per unit volume increases, which leads to the average distance available for dislocation motion becoming shorter. Henceforth, the dislocations encounter grain boundaries more frequently, resulting in greater resistance to plastic deformation. GBS therefore provides an effective method of increasing the strength of HEA without necessarily requiring the introduction of additional phases. This mechanism is particularly important in fine-grained HEAs produced through severe plastic deformation, thermomechanical processing, powder metallurgy, hot extrusion, or controlled recrystallisation.

#### 5.2.2. Dislocation Pile-Up and Grain Boundary Resistance

During plastic deformation, dislocations generated within a grain glide along their respective slip planes until they encounter a grain boundary, which represents a region of crystallographic discontinuity, causing the leading dislocation to be unable to freely transmit into the neighboring grain. Therefore, accumulation of additional dislocations occurs behind the leading dislocation, forming a blockage or pile-up of dislocations at the grain boundary region.

As dislocations accumulate, a local stress concentration begins to develop at the grain boundary, where the transmission of dislocations into the neighboring grain or the activation of an alternative slip system can occur once this stress reaches sufficient magnitude. Therefore, the applied stress required to initiate plastic deformation increases as the grain size decreases due to the shorter distance for development of dislocation pile-ups, leading to an increase in the frequency of grain boundary interactions [78,79].

#### 5.2.3. Hall–Petch Relationship and Grain Size Dependence

The quantitative contribution of grain boundary strengthening is commonly described using the Hall–Petch relationship (HP), where the yield strength of a polycrystalline material can be expressed by the formula, as shown below [78]:

(11) $$\Delta \sigma_{\mathrm{GB}}=k_{\mathrm{HP}}d^{-\frac{1}{2}}$$

where

Δ*σ_GB_*= the grain boundary strength; 

*k_HP_*= the Hall–Petch strengthening coefficient; 

*d*= the average grain diameter. 

Based on the relationship above, *σ_GB_* and *d* exist in an inversely proportional relationship, indicating an increase in GBS as the size of the grain decreases. This occurrence could be explained by the increase in the number of grain boundaries within the HEA structure as the grain size decreases, reducing the pathway for the propagation of the traversing dislocation effect. As the dislocations encounter grain boundaries more frequently, the stress required to be overcome for the propagation to continue to adjacent grains is increased, hence strengthening the HEA [80]. 

#### 5.2.4. Influence of Grain Boundary Character and Cohesion

Although the grain size is a significant parameter in influencing the GBS of HEAs, the effectiveness of grain boundary strengthening is not governed exclusively by grain size. The atomic structure and chemical state of the grain boundary also have the ability to strongly influence the transmission of dislocations and grain boundary cohesion within the HEA structure. A strong and coherent grain boundary could effectively accommodate dislocation accumulation and increase the stress required for slip transfer; however, a weak or poorly bonded boundary may undergo premature decohesion, which leads to a reduction in expected strengthening contribution.

This effect is particularly important in chemically complex HEAs, as the varying principal elemental segregation and chemical fluctuations within the HEA structure may alter the bonding characteristics of grain boundaries. Premature grain boundary decohesion in refractory HEAs has been demonstrated to reduce the accumulation of dislocations around the boundaries, resulting in lower measured stress than the results predicted by conventional strengthening calculations. In contrast, the addition of an element that has the property of enhancing grain boundary cohesion, such as boron, significantly promoted the accumulation of dislocations within the HEA structure, hence increasing the effective grain boundary strengthening response [81].

### 5.3. Phase Transformation Strengthening

Most conventional strengthening mechanisms operate by restricting the movement of dislocations, such as solid solution strengthening and grain boundary strengthening, as discussed in the preceding sections. Obstacles to dislocation motion are already established prior to deformation and will remain fixed throughout deformation. This creates a trade-off between a metal’s strength and ductility, where increasing the obstacle density would raise strength but simultaneously restrict the capacity for plastic deformation, which, in turn, reduces ductility. This remains one of the central challenges faced by engineers in materials engineering. With that said, phase transformation strengthening offers a fundamentally different approach by generating new microstructural barriers dynamically during deformation. This allows strength and ductility to be enhanced simultaneously. 

The stacking fault energy (SFE) is defined as the energy cost per unit area associated with a localized disruption in the regular atomic stacking sequence of an FCC crystal. It governs which deformation mechanism becomes active when the metal is under applied stress. The competing deformation mechanisms in the FCC phase, such as dislocation glide, twinning, and martensitic transformation, are determined by the intrinsic SFE [82]. Three main regimes are identified, as illustrated in Table 3 below [83]. 

In HEAs, the SFE can be deliberately tuned through compositional adjustments. For example, one can modify the element ratios or introduce additions such as Si, Al, or Mn, which shifts the SFE to selectively activate the desired deformation mechanism [83].

#### 5.3.1. Transformation-Induced Plasticity (TRIP)

In TRIP-active HEA, the FCC matrix is purposely engineered to be in a metastable state such that applied stress during deformation triggers a transformation into martensite. From this, two transformation pathways are possible:

1. FCC → BCC α’-martensite;
2. FCC → HCP ε-martensite.

The former is generally more beneficial, as α’-martensite is significantly harder and contributes more substantially to the strength–toughness balance [84]. As martensite forms at sites of the highest stress concentration, the newly created martensite–FCC interfaces act as planar barriers that continuously reduce the dislocation mean free path; a phenomenon known as the dynamic Hall–Petch effect [85]. 

This progressive, deformation-driven hardening is what distinguishes TRIP from static mechanisms: as the material keeps strengthening, the more it deforms rather than plateauing [17]. This was first demonstrated by Li et al. in a metastable dual-phase Fe_50_Mn_30_Co_10_Cr_10_ HEA, where the TRIP effect was shown to overcome the conventional strength–ductility trade-off through sustained phase transformation from FCC to HCP upon deformation [17].

#### 5.3.2. Twinning-Induced Plasticity (TWIP)

At higher SFE values within the intermediate regime (20–40 mJ/m^2^), deformation produces mechanical twins rather than a full phase transformation. In essence, a mechanical twin is a thin region of the crystal where the lattice is mirrored about a specific crystallographic plane, forming a coherent boundary with the surrounding parent matrix. These twin boundaries act as barriers to dislocation motion similarly to grain boundaries, which effectively subdivide the grain and reduce the dislocation mean free path. Notably, unlike grain boundaries, which are fixed prior to deformation, twin boundaries form continuously and progressively under load, meaning that the effective grain size keeps shrinking dynamically as deformation proceeds, allowing it to sustain high strain hardening rates over large plastic strains and postponing fracture [83]. This dynamic Hall–Petch effect is what enables TWIP alloys to maintain both high strength and high ductility simultaneously, as demonstrated in the Fe_50_Mn_30_Co_10_Cr_10_ HEA system where TWIP and TRIP effects were observed to operate concurrently depending on local SFE conditions [17,85].

#### 5.3.3. Combined TRIP and TWIP

With TRIP and TWIP introduced, the most mechanically exceptional HEAs are designed to activate both of these phenomena simultaneously within the same microstructure. This is achievable as HEAs possess local chemical heterogeneity, where compositional fluctuations between and within the grains create variation locally in the SFE, which causes some regions to fall within the TRIP regime and others to fall within the TWIP regime [83]. 

In the TWIP regime, coherent twin boundaries obstruct dislocations and promote pileups, whereas in the TRIP regime, the advancing martensite fronts send new dislocations into the FCC matrix, which further refines and enhances the mean free path and strain hardening [85]. The combined activation of both mechanisms produces an end material that is simultaneously stronger and more ductile than each mechanism alone could achieve individually [86,87]. This overcomes the classical strength–ductility trade-off.

### 5.4. Precipitation Strengthening

Precipitation strengthening involves the formation of fine, second-phase particles within the alloy matrix that act as obstacles to dislocation motion. Thus, deformation requires greater applied stress to occur, thereby strengthening the material [1]. Unlike the preceding section, precipitation strengthening is accomplished through a controlled heat treatment. The alloy is aged at a specific temperature and duration to nucleate and grow precipitates of a desired size and volume fraction within the matrix. In HEAs, this mechanism is particularly potent due to the sluggish diffusion effect discussed in the Core Effects section, which retards precipitate coarsening and enables fine nanoscale dispersions to remain stable, even at elevated temperatures where conventional alloys would lose their strengthening effect. [14].

#### 5.4.1. Dislocation–Precipitate Interactions

When a dislocation encounters a precipitate particle within the matrix, two competing mechanisms govern its response, depending on the precipitate size and coherency. In the shearing mechanism, the dislocation shears small, coherent precipitates, which disrupts the ordered internal structure of the precipitate and creates an antiphase boundary (APB). It is the energy cost of this disruption that resists dislocation motion and increases yield strength [88]. On the other hand, in the Orowan bypass mechanism, precipitates that are too large or hard to be sheared force the dislocation to bow around them. This leaves a dislocation loop that progressively tightens the spacing for any subsequent dislocations [88]. 

These two mechanisms define an optimal strengthening point at their crossover. Precipitates that are too small provide insufficient resistance, while coarse precipitates increase inter-particle spacing and reduce strengthening effectiveness [88]. Therefore, controlling aging temperature and the duration to target this optimal point is a critical processing consideration in the design of precipitation-strengthened HEAs.

#### 5.4.2. L1_2_ Precipitates

The L1_2_ ordered phase, which is structurally analogous to the γ’ (Ni_3_Al) phase found in nickel superalloys, is the most widely studied precipitate phase in FCC HEAs. In HEAs, L1_2_ precipitates of the Ni_3_(Al,Ti) type are induced through the addition of aluminum and titanium to CoCrFeNi-based systems. They form coherently within the FCC matrix [89]. This coherency between the precipitate and the matrix generates a misfit strain field that interacts with approaching dislocations from a long range, while cutting through the ordered precipitate interior creates an antiphase boundary (APB) that dislocations must travel in pairs to restore order. 

Both these effects contribute towards strengthening the alloy [90]. The strengthening effect is rather substantial. Studies have shown that adding Al and Ti to a CoCrFeNi alloy increases the yield strength from approximately 280 MPa to over 1000 MPa while retaining 17% ductility [89].

#### 5.4.3. B2 Precipitates

Apart from L1_2_ precipitates, B2-ordered precipitates, an ordered BCC derivative that is rich in nickel and aluminum, represent another crucial precipitate phase within HEAs, particularly in more Al-rich compositions.

Unlike L1_2_, which precipitates within grain interiors, B2 precipitates form preferentially along grain boundaries, pinning boundary migration and restricting grain growth during processing. This contributes to grain refinement strengthening alongside precipitation strengthening [91]. 

The combined existence of the intragranular L1_2_ and grain boundary B2 precipitates creates a multi-scale strengthening architecture that simultaneously strengthens both the grain interior and the boundaries. This was demonstrated in an Al_0.3_CoCrFeNi HEA, where the study showed that the introduction of both B2 and L1_2_ precipitates raised the yield strength from approximately 254 MPa to 710 MPa, all while maintaining ductility [91].

#### 5.4.4. Thermal Stability

A key advantage of precipitation strengthening in HEAs over conventional alloys is the superb thermal stability of the precipitates at elevated temperatures. Precipitate coarsening is driven mainly by atomic diffusion, meaning atoms must move for larger particles to grow at the expense of smaller ones through Ostwald ripening. As discussed in the Core Effects section, the sluggish diffusion effect in HEA significantly slows down this process, which enables nanoscale precipitate dispersions to remain stable at temperatures where conventional alloys would have coarsened and lost their strengthening [17]. This phenomenon has been proven through experimental means in high-entropy and superalloy systems. The experiment has demonstrated that stable γ-γ’ microstructures were maintained without forming deleterious TCP phases after prolonged exposure at 900 °C for 300 h [88]. 

This exceptional thermal stability makes precipitation-strengthened HEAs prime candidates for high-temperature structural applications where conventional Ni-based superalloys approach their performance limits [92].

## 6. Fabrication

In this section, the common fabrication methods of HEAs are described in detail.

### 6.1. Liquid-Phase Processing (LSP) Technique

The process of melting and casting using vacuum arc melting and mechanical stirring remains one of the most extensively used techniques in alloy development to fabricate HEAs [93]. The main challenges associated with the use of the LSP technique are the evaporation of low-melting elements during melting by using induction heating and resistance heating furnaces. Additionally, the challenges are attributed to the formation of heterogeneous microstructures when using LSP techniques due to the slow solidification rate during HEA casting. Nonetheless, LSP has been successfully implemented in several studies for HEA fabrication purposes [31,94,95,96,97,98].

#### 6.1.1. Arc Melting Technique

Arc melting is one of the main methods of manufacturing HEAs via liquid-phase processing, where elemental materials are melted using a high-temperature electrical arc. The electrical arc releases electric current between two electrodes, where the current generates high temperature in the region in which the arc is produced—this heats the metal precursors to form a molten pool [37]. The heat is maintained to enable the elemental material to melt and mix in a homogeneous manner. After the elements are mixed, the molten metal is allowed to cool and solidify in a mold to produce a desired HEA shape. Since molten metal reacts with oxygen at high temperatures, the mixing process is performed in an inert environment, such as argon gas, to prevent oxidation and contamination. 

The developing interest in research on using the arc melting technique in the production of HEAs is due to the lower porosity, low energy consumption, and time savings associated with using this method [93]. There are several advantages in using this method of fabrication for producing HEAs: one of the advantages is that the arc melting method enables a simple and relatively low-cost process compared to other fabrication methods [37]. It is also capable of melting multiple elements at different melting points, which makes this fabrication method suitable for producing HEAs. Moreover, the arc melting method is scalable since it can produce large-scale products at a fast rate, since the electrical arc produces high temperatures quickly. Several studies have indicated the use of the arc melting technique to manufacture HEAs with significant properties [99,100,101]. Otto et al. utilized the arc melting technique to fabricate an equiatomic CoCrFeMnNi HEA, where the investigation of the produced HEA at room temperature revealed a Vickers hardness of 4.2 GPa [95]. On the other hand, the arc melting method possesses several limitations, such as the production of heterogeneous microstructures due to how the mixing of the elements may not always be completely uniform, which can result in an uneven distribution of elements. The process consumes a large amount of energy, which is not environmentally friendly due to heat dissipation. Additionally, this method experiences uneven cooling after melting, which indicates that different regions of the alloy may vary in terms of their structure and properties. Microstructural variations can induce inconsistency in mechanical properties in terms of strength and ductility and form dendritic configurations [102]. Additionally, a study conducted by Ma et al. developed Al_0.5_CrCuFeNi_2_ HEA using the arc melting technique—the as-cast alloy possessed a simple FCC phase solid solution with a dendritic microstructure configuration [103].

#### 6.1.2. Induction Melting Method

The induction melting technique is a liquid-phase fabrication method that is used to produce bulk HEA. Unlike arc melting, which relies on using an electrical arc to provide a heat source, induction melting utilizes electromagnetic induction to generate heat inside the metal charge [93]. The magnetic field is produced when an alternating current passes through a copper induction coil. This magnetic field induces eddy currents, where the resistance of the metal to these currents generates heat that melts the elemental HEA powder placed in the crucible above the coiled wire. During melting, the HEA or ingot is placed inside the crucible furnace, which prevents the applied current from passing through it, causing the generation of magnetic fields, resulting in the melting of the HEA/ingot. The cool-down and remelting of the HEA/ingot is performed to produce a homogeneous cast sample of the HEA, where the melting process is conducted in a high vacuum. After all repetitions of cooling and melting are completed, the molten HEA is poured into the mold and allowed to solidify [104,105,106,107,108]. The induction melting technique is more advantageous than the arc melting technique due to its efficiency [93]. This technique is more environmentally friendly since it has lower energy consumption, as the heat produced is comparatively less than that of electrical arc. Research also indicates that this form of HEA production is more governable, where the microstructure of the HEA can be more homogeneously uniform [109]. Karlsson et al. performed a comparative study on AlCoCrFeNi HEA manufactured using induction melting and selective laser melting (SLM) [110]. The results indicate that the microstructure produced by induction melting produced coarser grains than SLM since the rate of cooling via induction melting was lower, which enables grain coarsening to be induced. Moreover, induction melting is preferred over the arc melting method since it produces more defect-free products and therefore can be used in aerospace components and medical implants where precision and purity are of utmost priority. A study conducted by Qiu et al. investigated the effects of Fe in FexCoNiCu HEA at various x values of 1.5, 2.0, 2.5, and 3.0. The study implemented the use of the vacuum induction melting (VIM) method for melting the raw materials. After melting, the raw materials were placed in a graphite crucible, where the HEA was poured into a copper mold and allowed to solidify. The study discovered that the Vickers hardness values were 2.21 times greater at x = 2.5 than at x = 1.5 due to the formation of more BCC phases in the HEA [111]. Another similar study, conducted by Kim et al., utilized VIM to manufacture Co_10_Cu_20_Mn_30_Ni_40_ HEA under an Ar inert environment. The study successfully achieved homogenization of the alloy by melting the master alloy for 6 h in a graphite crucible at 1373 K, after which the homogenized cast ingot was later rolled into 1.5 mm thick sheets at a reduction ratio of 75%. The rolled sheets were then heat-treated at five different temperatures ranging from 873 K to 1173 K, where the sample treated at 873 K achieved the highest ultimate tensile strength value of 996 MPa [112]. Collectively, the key findings indicate that the use of VIM allows accurate control of heating and cooling rates to achieve homogeneity of the HEA compared to the arc melting method. The use of a vacuum environment prevents oxidation but presents the need for secondary machining to give a good surface finish [113]. Other limitations include the need for proper crucible selection—the melt produced by induction melting can react with the ceramic or refractory crucible, which can potentially introduce contaminants or alter the melt chemistry, especially in Ti-based systems [114]. Additionally, the production of large-scale and high-quality castings is challenging when using VIM since thermal gradients, solidification time, segregation, and inclusion control become increasingly challenging as the ingot size increases [115]. Other limitations include the high cost of equipment required to perform induction melting, such as a high-frequency power supply, induction coil, water cooling system, refractory/crucible system, etc., with additional costs for vacuum chambers and pumping systems for the VIM method [115]. The process is also energy intensive for VIM since it encompasses several processes such as evacuation, heating, melting, superheating, holding, pouring, cooling, and chamber turnaround—a study on application of VIM in producing Inconel 718 indicated that crucible choice and melting practices can have a significant effect on energy consumption and the total processing time [115].

#### 6.1.3. Bridgman Solidification Technique

The Bridgman solidification technique is a specialized liquid-processing method utilized to manufacture single-crystal or directionally solidified HEA. The Bridgman technique enables researchers to control the direction of crystal growth to produce highly controlled microstructures. This technique works by introducing a seed crystal into a crucible filled with molten material, which enables the molten material to cool, producing a unidirectional single-crystal polycrystalline alloy [116]. This technique is used extensively in manufacturing alloys to produce a specific grain configuration or alignment. One example of grain configuration is the columnar grain structure, which can be produced with a unique grain direction that is parallel to the axis of the crystal. The Bridgman technique controls grain growth alignment by controlling the thermal flow route, temperature gradient, and drawing speed of the solid–liquid interface during solidification to improve uniform cooling gradient and the properties of the alloy [117]. Ma et al.developed a single-crystal CoCrFeNiAl_0.3_ and CoCrFeNiAl HEA using the Bridgman solidification technique [118]. A study conducted by Zhang et al. conducted a comparative study on the effects of solidification techniques on the microstructure and mechanical properties of AlCoCrFeNi HEA—the techniques in this study were copper mold casting and Bridgman solidification methods, in which both methods produced stable BCC solid solution phases. X-ray diffraction (XRD) analysis was implemented to analyze the microstructure produced by the two distinct techniques—the analysis revealed that the copper mold casting technique produced a heterogeneous dendritic structure, which was characterized by the formation of flowery dendrites, columnar dendrites, and fine equiaxed grains due to uncontrolled heat extraction and fluctuating temperature gradients. On the other hand, the Bridgman solidification technique produced a microstructure that exhibited a more uniform equiaxed grain structure, where this difference in morphology was attributed to the higher temperature-to-growth rate ratio, which reduced constitutional supercooling and promoted stable grain growth. Key findings indicated that the Bridgman-solidified samples exhibited enhanced plastic deformation capability, with an increase in the maximum plastic strain from 21.6% in its as-cast condition to 29.5% at a withdrawal velocity of 1800 μm/s. This represented a plasticity improvement of approximately 35% [119]. Similarly, Zuo et al. produced FeCoNiAlSi HEA using this method as well. The comparative study between the arc melting method and Bridgman solidification technique concerning HEA properties revealed that more favorable characteristics (such as magnetic properties) were produced when using the Bridgman solidification method [119]. This form of manufacturing HEA is observed to be applied in the manufacturing of optical components due to their nature of low absorptivity and high transmissivity. On the other hand, the Bridgman solidification method poses several limitations, where impurities can be introduced as the growing crystals remain in contact with the crucible [120], wall-induced nucleation occurs, and non-equilibrium solidification produces dendritic micro-segregations, which may require homogenization heat treatment [121]. 

### 6.2. Solid Phase Processing Technique

#### 6.2.1. Mechanical Alloying and Conventional Powder Metallurgy

Mechanical alloying (MA) is a process in which HEAs are produced without needing the complete melting of their constituent elements [37]. This process involves placing elemental powders in a high-energy ball mill where the repeated collisions between the balls and the elemental powders induce severe plastic deformation and elemental diffusion between particles. The continuous impact causes the powder particles to undergo cold welding, fracturing, and re-welding that induces atomic-level mixing and formation of a homogeneous alloy structure [94]. 

Apart from using particle size reduction methods, this technique can be integrated to increase the strength and hardness of the alloy. Additionally, MA causes the breakdown of particles, which results in the production of bulk mass with magnified porosity. MA induces the alloyed materials to have low densification, high porosity, and low strength [122]. Hence, conventional powder metallurgy techniques that involve compaction and sintering are employed to enhance the properties of the alloyed materials by increasing their density, porosity, and strength; this is achieved by integrating a process known as sintering, where the bulk material is compressed and heated. Prior to the compression of the bulk material, binding agents and lubricants are added to the ball-milled elemental powders to promote the bonding of the particles. Next, the powders are heated to an increased temperature, which induces solidification and densification of the bulk mass. As a result, the bulk HEA possesses high strength, higher density, and lower porosity and defects. Conventional sintering has been extensively used for several decades since it is simple, cost-effective, and scalable [37]. One of the drawbacks of conventional sintering methods is that it is time-consuming and does not produce perfect densification. Porosity affects the strength and other properties of its products. Ruiz-Esparza-Rodríguez and colleagues compared two different sintering methods in processing AlxCoCrFeMnNi HEA after performing MA—the two methods were high-frequency induction heating and conventional sintering. The results indicated that the densification produced by high-frequency induction heating is comparatively higher (95%) than its conventional sintering counterpart (80%) [123]. Moreover, sintering through induction heating produced a comparatively higher microhardness due to more refined grains [37]. However, long milling times, contamination and oxidation of fine powders, and residual porosity or grain coarsening during conventional sintering remain important limitations.

#### 6.2.2. Non-Conventional Sintering

Non-conventional sintering is an advanced powder consolidation process used to produce HEA by means in which heat is not directly supplied to the blended elemental powder sample for atomic diffusion inducement [37]. This process uses alternative heating methods and external energy sources to enhance the densification process. Heat energy, in the form of electromagnetic radiation, laser, or pulsed direct current, is directed to the blended powder, which results in atomic diffusion; this induces the formation of strong bonds between the elemental powders [124]. This setup is shown below.

Unlike conventional sintering techniques that rely on furnace heating, non-conventional sintering provides rapid heating and hence shorter processing times, which enable it to be more efficient in producing HEAs with superior properties using less energy consumption [125]. Since this method induces heating and cooling rapidly, there is not sufficient time for grain coarsening to occur. The extreme heat produced in this process has demonstrated the ability to vaporize impurities to produce products with lower impurities and lesser porosity [126]. Research has indicated that non-conventional sintering methods perform better than their conventional counterparts. One of the most widely used examples of non-conventional sintering methods is known as Spark Plasma Sintering (SPS), where a pulsed direct current and external pressure are applied to the powder compact, which causes significant heat to be generated, causing the powders to form, which induces high densification. The bulk mass-produced material exhibits enhanced microstructures with zero grain coarsening, interlocked grain-boundary lines, a cohesive matrix/additive interface, uniformly distributed reinforcements, and the absence of pores. Kumaran and colleagues performed a comparative study between the use of SPS for commercial commodity alloy powders and conventional pre-alloyed or mechanically alloyed powders using three non-equiatomic CoCrFeNiMo_x_ HEAs (C1, C2, and C3). Microstructural analysis revealed that the SPS process was able to maintain a fine-grained structure due to the rapid heating rate and short holding time. The grain size remained significantly smaller compared to equivalent HEA produced by conventional casting methods, which indicates the advantage of using SPS. High-temperature compression testing revealed that SPS-produced HEAs exhibited excellent mechanical performance at elevated temperatures. Notably, the C2 alloy exhibited the highest compressive strength of 1352.5 MPa at room temperature and maintained 642.3 MPa at 750 Celsius, with a ductility above 44% [127]. The excellent mechanical performance at high temperatures was attributed to the stable FCC structure and molybdenum (Mo) content that induced solid solution strengthening. The key findings indicate the potential of using SPS in producing HEA directly from comparatively low-cost commercial powders, which rids the need for pre-alloying and improvisation of HEA composition design. A study conducted by Yadav et al. investigated the use of SPS to fabricate HEAs using a non-conventional powder metallurgy route. The study highlighted the advantage of using SPS compared to conventional sintering methods due to its ability to achieve high densification with limited grain growth due to its rapid heating rate, short holding time, and applied pressure. The study also indicated that the use of SPS enabled phase transformation control, where metastable FCC or BCC solid solution phases in mechanically alloyed powders could transform into single-phase or multiphase structures—the ability to configure phase formation enables control over the strength, hardness, ductility, and thermal stability of HEAs. In the study, the use of SPS on CoCrFeMnNi HEA produced an FCC structure with a tensile strength of approximately 1000 MPa at 6% ductility—values that are comparatively higher than the equivalent cast alloy. Additionally, the study proved that SPS could retain grain structures and allowed the integration of strengthening mechanisms such as grain boundary strengthening, solid solution strengthening, precipitation strengthening, and dislocation strengthening. The study produced Co_25_Ni_25_Fe_25_Al_7.5_Cu_17.5_ HEA using an MA and SPS route, where the yield strength of 1795 MPa and 454 HV hardness were achieved [128]—a significant improvement compared to the cast alloy. On the other hand, the limitations of SPS include difficulty of scaling up and the high costs of equipment. SPS requires the use of specialized equipment and precise parameter control, which can incur high processing costs compared to its conventional sintering counterparts. The requirement of controlled environment/vacuum systems, conductive dies, and high-pressure systems increase the manufacturing cost and complexity, which poses a significant challenge for its application at an industrial scale.

### 6.3. Gas-Phase Processing Technique

#### 6.3.1. Sputtering

Sputtering can be defined as a physical vapor deposition technique used to produce HEA films where particles of gas and plasma possessing high kinetic energy are bombarded onto the surface of a solid material, resulting in the release of atoms that are ejected and deposited from the surface. This method is commonly used in the deposition of thin films and etching of metals, manufacturing of optical coatings, semiconductors, and nanotechnology components [125,129]. The use of sputtering can be applied to the manufacturing of HEAs by depositing multiple elements simultaneously onto a substrate. In this process, a solid HEA target material is placed inside a vacuum chamber containing inert gas, where a high voltage is applied to generate plasma, which causes the inert gas ions to accelerate and collide with the target forces; the transfer of kinetic energy causes the atoms to be ejected, where they are deposited onto a substrate to form a thin layer of HEA coating [130]. The main types of sputtering methods used for HEA fabrication are direct current (DC) and radio frequency (RF) magnetron sputtering techniques. A study conducted by Liao and colleagues utilized the magnetron sputtering deposition technique to produce high-entropy alloy thin films (HEATFs) at 4 μm of thickness. This method enabled the production of smooth, homogeneous nanocrystalline HEA coatings. The nanocrystalline structure featured an FCC-dominant phase, which achieved a high yield strength of 1024 MPa and hardness of 11.09 GPA; the values are approximately three times higher than those of its bulk counterparts [131]. In the sputtering method, one of the sources of defects is derived from the high mechanical energy of ejected elements, which results in heavy plasticity in the deposited material. Sputtering offers a wide range of advantages, such as being environmentally friendly, as the process occurs at low temperatures. The process is also easily scalable, which enables this process to be a practical method that can be implemented in industry. The limitation of the sputtering technique is the difficulty of applying this technique to complex geometric devices due to its “line-of-sight” process. Moreover, the use of the magnetron sputtering technique is only limited to thin-film coating applications rather than bulk material fabrication. Other drawbacks include the high cost of the equipment and the time-consuming process.

#### 6.3.2. Molecular Beam Epitaxy

Molecular beam epitaxy (MBE) is an advanced gas-phase physical vapor deposition method used for producing films of crystalline substances and nanostructured HEAs in a vacuum. The process involves directing rays of atoms to a substrate heated to a temperature significantly above its melting point; this results in the deposition of atoms onto the surface of the melting substrate [132]. Deposition occurs as the atoms condense onto the surface of the substrate in a strata-by-strata configuration to form a single-crystal film; this is known as epitaxy [133]. MBE is performed inside a vacuum chamber that is equipped with multiple high-precision spectrometers and crystal monitors, which allow the accurate control of the composition and thickness of the film being deposited. The crystal film formed follows the same crystal configuration as the substrate—this feature enables MBE to be used in the development of complex configurations such as quantum wells and superlattices in semiconductors [134]. The process occurs at a low temperature, which prevents defects associated with high temperatures. MBE enables precise control over the composition and weight of the film deposited on the substrate. Hence, MBE is applied to industries such as semiconductors, photonics, and optical devices. Studies have indicated the use of MBE in preparing compositionally graded Fe-Ni-Co-Cu-Ga HEA thin films. The deposited films with differences in composition across the substrate were annealed and analyzed using Energy Dispersive X-Ray Spectroscopy (EDS), Scanning Electron Microscopy (SEM), and XRD methods [135]; the study revealed that MBE can be used with high-throughput gradients to swiftly screen HEA compositions in a single substrate. This finding highlights the reduction in time required to analyze composition–structure–property relationships. Another study investigated the application of MBE to grow rare-earth HEA thin films of a DyGdHoLuTb composition on a niobium buffer layer with controlled crystal orientation and random elemental distribution. With this, these methods enabled research to study the magnetic behavior of polycrystalline materials in the absence of grain-boundary effects [136]. Although both MBE and sputtering are forms of physical vapor deposition processes, their processes are different from one another. MBE involves the deposition of atoms onto a substrate via a high-energy beam, while sputtering is the ejection of particles or atoms from the source due to the bombardment of gas/plasma particles on the surface of solid material. The working principle of MBE enables smoother film deposition compared to the sputtering technique [86]. MBE can also be used to coat complex contours in ways that sputtering cannot. MBE forms a single-crystal film at once, while sputtering relies on a layer-by-layer process to form the crystal film. MBE requires a vacuum during its operation, while sputtering does not. The use of MBE enables atomic- or monolayer-scale thickness control, and MBE can produce highly uniform, low-defect semiconductor films when substrate, flux, vacuum, and temperature are well controlled. However, MBE is limited by its intrinsically low deposition rate, high capital, and operating costs associated with the ultra-high vacuum infrastructure and high-purity source materials. Moreover, high-quality MBE requires strict control of the source flux, substrate temperature, beam composition, chamber contamination, and shutter timing. Lattice mismatch and substrate defects may give rise to dislocations, roughness, cracks, and other defects [137]. Additionally, MBE is suitable for thin, high-value epitaxial layers but is generally unsuitable for thick films and low-cost, high-volume manufacturing.

### 6.4. Thermal Spraying

Thermal spraying can be termed as a surface coating fabrication technique used to deposit HEA coatings onto the substrate with a spray gun in the form of powder, rod, or slurry. Thermal spraying is executed with the purpose of improving surface properties of the coated substrates to increase wear resistance, corrosion resistance, oxidation resistance, and thermal stability [138]. 

The process involves heating the feedstock to its melting point, where it is accelerated towards the substrate surface. There are three distinct types of thermal spraying methods: high-velocity oxygen fuel (HVOF) spray, heating from electric plasma or arc, atmospheric plasma spray (APS), heating from low-temperature sources, and gas expansion using the cold spray (CS) method [139,140]. Xinqing Ma et al. devised a NiCoCrAlSi HEA composite coating on a metallic substrate using the HVOF and APS methods [141]. The study also observed that the type of spraying method used affects the phase formation—HVOF produced a single BCC phase, while APS produced BCC, B2, and L12 phases. Another study on different types of feedstock preparation methods of AlCoCrFeNiTi HEA revealed that different preparation methods influenced the hardness due to the microstructure formed. The study investigated APS spraying in three preparation ways: blending of powder, high-energy ball milling, and gas atomization of the powder. The study found that gas-atomized feedstock has the highest hardness; the gas-atomized feedstock also had the finest microstructure [142]. The advantage of thermal spraying is the fast process time while offering a range of coating thickness values from 100 mm to a few mm [143]; it is affordable, operates at low temperatures, and does not threaten the environment. It works on a range of materials such as ceramics, alloys, HEAs, polymers, and composites [139]. The limitation of thermal spraying lies in the lack of smooth finishes and can be affected by porosity and the presence of impurities. This technique is used in industries, citing its scalability, environmental friendliness, and affordability. Additionally, conventional thermal-sprayed coatings commonly possess a lamellar splat-based microstructure that contains pores, oxide films, unmelted particles, intersplats, and microcracks; these features reduce cohesion, induce anisotropic properties, and allow corrosive media to reach the coating–substrate interface. The process also requires direct line of sight, making it unsuitable for use in deep internal surfaces and geometrically complex and recessed regions [144]. Moreover, coating quality is highly dependent on feedstock, substrate preparation, particle temperature, particle velocity, spray angle, stand-off distance, and deposition strategy [145,146].

### 6.5. Laser Cladding

Laser cladding is an advanced surface-modifying process that uses a laser beam to fabricate HEA coatings on materials such as metals, alloys, composites, and polymer substrates to improve surface properties such as wear resistance, corrosion resistance, and high-temperature performance [37]. The process involves the use of a laser pointed at the substrate, where the heat emitted from the laser melts the material being deposited and joins onto the substrate to form a coating layer. 

Guo et al. utilized laser cladding methods of CoCr_2_FeNiTi_x_ HEA supported with TiN on a 904 L Stainless steel substrate [147]. The study revealed that the hardness of the cladded HEA-TiN was approximately three times higher than the hardness of the substrate; the wear mass was 0.333 times that of the substrate, with a significant reduction in corrosion resistance. Another study investigated the use of powder and wire laser cladding, which successfully produced CrMnFeCoNi and Al_x_CoCrFeNi coatings with high chemical and structural homogeneity. In the *Al_x_*CoCrFeNi coating, the increase in Al content induced a transition from an FCC solid-solution structure to a BCC structure at Al contents above approximately 15%; this resulted in an increased hardness value from 200 HV to 600 HV [148]. The study also reported an achievement of approximately 100% material efficiency. Another study on TaNbZrTi refractory HEA coating was fabricated using multi-channel laser cladding; the coating consisted of BCC_1_, BCC_2_, and Fe_2_Nb phases. The computed theoretical yield strength equated to approximately 1816.1 MPa, with strengthening contributions from various strengthening techniques employed [149]. The key findings indicate that laser cladding produced HEA can achieve high strength, where the importance of substrate dilution and high-temperature oxidation must be controlled properly. Another study performed on HEA coatings deposited onto Cr12MoV die steel discovered that increasing the laser power from 1400 W to 1700 W led to an increase in the molten-pool peak temperature from about 2005.5 Celsius to 2357.4 Celsius [131]. The study also determined the optimal laser power at 1600 W, which produced an average microhardness of about 600 HV, a value 150% higher than the 240 HV substrate hardness. Hence, this study highlighted the importance of selecting the optimal laser power to maximize the mechanical performance of the HEA coatings. Unlike thermal spraying, laser cladding offers higher accuracy and more control, and enables less damage to the substrate [150,151]. Laser cladding, however, is more costly than thermal spraying, where the materials that are to be deposited must be carefully selected. The advantages of laser cladding are the formation of stronger metallurgical bonding between the HEA coating and substrate, a localized heat-affected zone, and little distortion of the substrates [152]. On the other hand, laser cladding is limited by strong thermal gradients, rapid solidification, and differential thermal contraction between cladding material and substrate—these effects produce residual stresses, which can induce distortion, microcracking, solidification cracking, and reduced fatigue or corrosion performance. Moreover, the process requires high-cost equipment, skilled operation, restricted line-of-sight access, and frequent post-processing [153,154,155].

### 6.6. Electrodeposition

Electrodeposition is defined as an electrochemical fabrication technique used to deposit HEA coatings on the surface via an electric current, which causes oxidation at the anode and a reduction reaction at the cathode within the electrolytic system. The tank contains two distinct electrodes known as the cathode and anode—when a potential difference is set, the oxidation reaction occurs at the anode while the material that is to be deposited undergoes a reduction reaction at the cathode [156,157]. This process is used for producing thin coatings and surface layers featuring improved corrosion resistance, wear resistance, high thermal and electrical conductivity, and is used to enhance its esthetic appeal. The amount of deposited substance is determined by variables such as the voltage supplied, current, time, concentration of the electrolyte, etc. Research conducted by Popescu et al. studied electrodeposition to deposit CoCrFeMnNi HEA onto copper substrate in dimethyl sulfoxide electrolyte solution; the study discovered that the deposited films possessed high corrosion and wear resistance of the deposited HEA film [158]. Another study conducted by Freitas et al. investigated the electrodeposition of CoFeNiMoW HEA thin films using a citrate-containing electrolyte solution [159]. The advantages of using electrodeposition are the control of thickness and composition of the coating [160], cost-effective processes, and the low cost of its products [161]. The process is compatible with a wide range of material types such as plastics, metals, rubbers, ceramics, and more [162]. Additionally, electrodeposition can be used on shapes that possess complex and intricate geometries [163]. On the other hand, the limitations of electrodeposition include the tendency to introduce pores and defects [164], the process is time-consuming, and the challenges attributed to electrodepositing on alloys that have low melting points [165].

### 6.7. The Effect of Fabrication Route on the Final Phase Constitution of HEAs

HEAs are often designed according to nominal elemental composition. However, the composition alone does not fully dictate the final phase constitution or mechanical behavior of the HEA. Such characteristics are determined by the fabrication route, which controls the thermal cycle, cooling rate, diffusion time, elemental partitioning, grain size, defect population, density, and residual stress. Fabrication routes dictate the final phase constitution, grain morphology, cellular structures, and microscale chemical segregation, which have a profound impact on the alloy’s corrosion, resistance, fatigue behavior, and creep performance [164].

Of the various fabrication routes, the melting and casting fabrication route stands as the most extensively used and cheapest fabrication technique [47]; this method, however, has a high tendency to produce heterogeneous structures, elemental segregations, and defects [166]. PM consisting of MA coupled with SPS is a fabrication route chosen to produce more homogeneous microstructures in HEA; the process is quick and both resource- and energy-efficient. However, the PM fabrication route faces challenges attributed to impurities stemming from the grinding media when producing HEAs in such a manner [167]. In contrast, the recent growing interest in incorporating the use of AM techniques for synthesizing HEAs has highlighted the flaws of the other mentioned fabrication routes.

#### 6.7.1. Final Phase Constitution of HEAs Synthesized Using Melting and Casting Fabrication Route

Conventional melt-processing techniques, which include arc melting, induction melting, and vacuum melting, are widely employed in the production of bulk HEAs due to their ability to manufacture dense and relatively large components [168]. However, the broad solidification ranges and complex elemental partitioning nature of HEAs can induce microsegregation, the formation of interdendritic intermetallic phases, and in extreme cases, hot cracking. An example is an AlCoCrFeNi HEA manufactured using melting and casting methods, which exhibited BCC+B2 phases with dendritic microstructures [169,170]. Tian et al. [171] investigated the effects of different cooling rates when using the arc melting fabrication technique on AlCoCrFeNi HEA—the study observed the presence of B2 phases in the nanoparticles within the grains of the single-phase BCC structure. It is imperative to note that the phases that form in HEAs are more influenced by the interactions between pairs of elements rather than viewing them individually [172]. One example of this is the AlCoCrFeNi alloy, which forms a BCC phase structure even though only Cr and Fe are BCC crystal structures when compared to its overall constituents. The AlNi pair serves as the primary crystal structure due to the similar lattice parameter between AlNi and AlCoCrFeNi [173,174]. The AlNi binary pair can dissolve other constituent elements [175,176], resulting in other elements dissolving into it due to its chemical compatibility and mixing entropy of the multicomponent alloy [177].

Examples of HEA systems studied that exhibit single-phase FCC phases using melting and casting techniques include Al_x_CoCrCuFeNi [178,179], CoCrCuFeNi [180,181], FeCoNiCrCuO_0.5_Al_x_ HEA [182], and Al_x_CoCrFeNiTi_y_ HEA [183,184]. The formation of the FCC phase is affected by the interactions between the constituent elements within these alloys. Elements such as Cu and Ti can further promote the stabilization of the FCC phase [185,186]. In the Al_x_CoCrFeNi system, Ti addition has been reported to induce the phase evolution from a predominantly BCC structure to an FCC structure [187]. In a similar case, replacing Al with Cu in AlCoCrFeNi to give CoCrCuFeNi promotes the formation of an FCC phase rather than an A2 + B2 structure, which is typically associated with the Al-containing alloy; such behavior can be attributed to the FCC phase-forming tendencies of binary combinations, such as Cu-Co, Cu-Ni, Co-Ni, Fe-Ni, and Co-Fe present within the CoCrCuFeNi system. Furthermore, the incorporation of Mn into CoCrFeNi to produce CoCrFeMnNi also favors the formation of a single-phase FCC structure [188].

Another study investigated the effect of cooling rates on the microstructure of Al_x_CoCrFeNi HEAs using arc-melting and suction casting techniques—the study discovered that higher cooling rates in suction casting resulted in more refined columnar dendrite grains [189]. Both processes resulted in the formation of BCC and FCC phases, with a B2 phase included for arc melting and a Laves phase for suction casting. Therefore, quicker cooling rates tend to promote the formation of a dominant, single phase while suppressing the precipitation of secondary phases [190]. Rapid solidification techniques, which encompass melt spinning, splat quenching, and laser remelting, can suppress elemental partitioning, hence promote the formation of supersaturated solid solutions and refined microstructures [168]. Directional solidification and controlled cooling have been used to produce eutectic HEAs where lamellar B2/L1_2_ or FCC/BCC architectures enable the microstructural homogeneity of the HEA, providing a balance between strength and ductility. Recent studies have indicated that directional solidification coupled with thermomechanical processing can modify lamellar spacing and phase connectivity, resulting in improved fracture resistance of the alloy.

Eutectic HEAs are a specialized class of HEAs formed from the solidification of a eutectic reaction, resulting in the formation of two or more distinct solid phases within a lamellar structure or composite microstructure. Unlike conventional single-phase HEAs, eutectic HEAs utilize a multiphase structure to achieve a balance between their mechanical properties; they combine strength and ductility phases to do so. The B2 phase (BCC phase ordered) is typically hard and brittle, while the L1_2_ phase (FCC phase ordered) is ductile and tough. The combination of FCC and B2 phases enables such balance. One example of the FCC/BCC architecture is the AlCoCrFeNi_2.1_HEA, which consists of B2//L1_2_ phases [191]. Other phase pairings of eutectic HEAs include FCC/B2 (or FCC/BCC), BCC+ intermetallic (σ-phase or µ-phase) [192], and FCC+ Laves phases [193]. 

Nonetheless, the primary challenges in using melt-based fabrication routes for synthesizing HEAs are namely: (a) controlling the thermal gradient and cooling rate to prevent the formation of undesirable phases; (b) controlling macrosegregation in large castings; and (c) developing suitable post-cast homogenization treatment to reduce undesirable interdendritic phases while preventing excessive grain growth [194,195,196].

#### 6.7.2. Final Phase Constitution of HEAs Synthesized Using Powder Metallurgy, Mechanical Alloying and Sintering Fabrication Routes

Solid-state fabrication routes comprising MA, PM, and sintering techniques (like SPS) have been used to synthesize nanocrystalline HEAs [167,197,198]. The use of PM coupled with MA and SPS for producing HEAs has been documented in various studies. MA is a process capable of producing ultrafine-grained or nanocrystalline microstructures that contribute to the mechanical strength through grain refinement and solid solution strengthening [199]. The refined microstructures enhance the hardness, enabling these alloys to be used for wear-resistant applications. Despite their high strength capabilities, some of these alloys retain their ductility and toughness, which can be further optimized by implementing post-milling treatments such as annealing [47]. Their good corrosion resistance can be attributed to the uniform distribution of alloying elements and the ability of elements such as Cr, Al, and Ti to promote the formation of stable passive layers. Mechanically alloyed HEAs have shown potential for high-temperature applications where resistance to creep and thermal degradation is paramount.

HEAs produced through MA exhibit considerable functional versatility due to the ability of the process to tailor their composition and microstructure. Certain Ti-, Zr-, and V-containing HEAs exhibit favorable hydrogen absorption and desorption kinetics, making them a promising candidate for hydrogen storage applications [200,201,202,203]. Fe-Co-Ni-based HEAs also demonstrate tunable magnetic properties, which are of interest for energy and electronics applications. The uniform atomic-scale mixing can minimize elemental segregation and promote the formation of solid-solution or amorphous structures. These microstructural characteristics, with the refined grain structure produced during milling, contribute to the improved fatigue resistance and durability under cyclic loading. Overall, the ability to alter the phase constitution, microstructure, and functional properties through MA highlights the potential of MA-derived HEAs in applications ranging from energy storage and conversion to applications for structural components.

Binary-phase diagrams of constituent elements, together with the thermodynamic mixing enthalpy of a HEA system, can provide useful insight into the phases that may develop during MA [204]. The energy introduced during high-energy milling generates localized heat, which can affect the phase evolution of the alloy. Typically, the natural tendency of alloy systems is to favor phases of lower Gibbs free energy; it is therefore crucial to control MA parameters to obtain the desired phase constitution. Important parameters of MA include the milling duration, ball-to-powder ratio (BPR), and the type of milling media used. Chen et al. [205] investigated the alloying behavior, microstructural evolution, and mechanical properties of FeNiCrCo_0.3_Al_0.7_ HEA produced via MA and SPS. After a duration of 45 h of milling, the alloy produced a refined and chemically homogeneous supersaturated BCC solid-solution structure. During the initial 6 h of milling, Al and Co dissolved into a solid solution more rapidly than other constituent elements due to their comparatively low melting temperatures, which aided their diffusion during the milling process despite their lower concentrations in the alloy. As MA progressed, elements such as Cr and Fe gradually dissolved into the solid solution. Since both elements possess a BCC structure, their lattices can accommodate additional alloying elements with relatively limited lattice expansion. However, Cr possesses the highest melting point among the constituent elements and therefore has comparatively slower diffusion during milling. The repeated cold welding and fracturing during MA progressively refine the crystallite size. In addition, the difference in the atomic size among the constituent elements induces lattice distortion within the HEA. Prolonged milling further increased the dislocation density and lattice strain due to severe plastic deformation, where crystallite refinement increases the proportion of grain boundaries.

A study conducted by Vaidya et al. [167] investigated the influence of elemental addition sequence on the phase evolution of nanocrystalline AlCoCrFeNi HEAs produced by MA. The study consisted of performing sequential addition of remaining elements into different binary systems representing B2 (AlNi, AlCo, and AlFe), BCC (FeCr), and FCC (CoNi and FeNi) structures. The result indicated that both the initial phase and the order in which the elements were introduced affected the phase evolution. For instance, the addition of Ni to an AlCo-based B2 structure promoted the development of an FCC solid solution rather than maintaining the B2 structure. Subsequent additions of Fe and Cr destabilized the FCC phase, resulting in a final microstructure that is predominantly BCC with smaller FCC fractions. Similar BCC-FCC phase evolution was reported for mechanically alloyed CoCrFeNi alloys [206,207].

Interestingly, the final BCC + FCC microstructure of AlCoNiFeCr remained relatively consistent despite changes in the elemental addition sequence and milling duration. However, arc-melted AlCoCrFeNi is reported to form predominantly single-phase BCC structures [208]. Nonetheless, the results indicate that the initial binary structure, elemental addition sequence, milling conditions, and elemental interactions can all influence the phase evolution during MA.

Ni and Co generally promote the FCC formation, although Co can preferentially interact with Al when introduced immediately after it. In contrast, Al tends to stabilize the BCC phase, while Cr also favors BCC formation [4,209]. Fe has comparatively weaker influence on phase selection due to its less negative mixing enthalpy and smaller atomic radius. The formation of supersaturated BCC solid solutions during MA has been associated with the high energy stored at grain boundaries, increased mixing entropy, and extended solid solubility. However, the metastable phases produced during milling can transform during subsequent SPS consolidation. An example is the partial BCC phases in FeNiCrCo_0.3_Al_0.7_ that were reported to transform towards the more stable FCC phase after SPS at 1000 °C [205,210]. This transformation is attributed to the recovery and annihilation of defects introduced during high-energy milling, allowing atomic rearrangement at elevated temperatures. Similarly, Ji et al. [198] obtained a BCC solid solution in CoCrFeNiAl after 60 h of MA, followed by the formation of BCC + FCC phases after SPS at 900 °C.

Milling parameters can also influence phase formation [211]. Kumar et al. [212] produced a supersaturated BCC solid solution in AlCoCrFeNiSi_x_ after only 20 h of milling at 300 rpm, whereas a study conducted by Ji et al. [198] required 60 h at 250 rpm to obtain a comparable BCC solid solution. This difference indicates the influence of milling speed on phase evolution. According to a study performed by Daryoush et al. [211], higher milling speeds induced more intense collisions between balls and powder particles, which accelerates the milling process to produce finer particles and enhances uniform mixing. However, extremely high milling speeds cause elevated temperatures in the milling chamber, which can potentially cause unwanted thermal effects. The ball-to-powder ratio can influence the final microstructure of the alloy. The ball-to-powder ratio is termed as the ratio of the mass of the milling balls relative to the mass of the powder mixture. The study discovered that higher ratios enhance the intensity of the milling process, leading to the enhancement of homogeneity, reactivity, and the production of finer particle sizes; on the other hand, significantly high ratios can result in excessive wear of the milling container and balls, which causes more frequent replacement of this equipment after use. The use of process control agents (PCAs) can alter the milling environment, improve powder flowability, prevent agglomeration, and reduce the tendency of powders to adhere to the vials or milling balls. Hence, the use of PCAs can significantly affect the alloying behavior, milling kinetics, and final characteristics of the synthesized materials. Therefore, milling parameters play an influential role in the final phase constitution of HEAs.

#### 6.7.3. Final Phase Constitution of HEAs Synthesized Using Additive Manufacturing

The advent of additive manufacturing (AM) has emerged as one of the most important fabrication processes for HEAs, citing its high design flexibility and significantly high localized cooling rates [47]. The rapid heating and cooling cycles when using AM generate new layers from thermal contraction [213]. The successive layer-by-layer deposition of AM enables the production of microstructures that are not possible when using conventional melting and casting techniques. Moreover, the layer-wise buildup of AM produces a microstructure that is governed primarily by both rapid and directional solidification. The final phase constitution and microstructure are influenced by several process parameters, such as powder flowability, laser power, layer thickness, particle morphology, scan spacing, and volumetric energy density (VED). Hence, it is crucial to control these process parameters to regulate the microstructural evolution and synthesize HEAs with desirable properties. 

Some examples of HEAs synthesized using AM are AlCrFeCoNi HEAs, where studies conducted by Fujieda et al. [214] and Kuwabara et al. [215] have produced AlCrFeCoNi using selective electron beam melting (SEBM). The microstructure of the HEA produced by AM exhibited a BCC + B2 phase constitution that was the same as when using conventional melting and casting methods despite significant differences in solidification rates between the two distinct fabrication methods [171,216]. However, an additional FCC phase was discovered near the bottom of the SEBM-built material; this is potentially due to the transformation of BCC/B2 phases at a lower temperature that occurs during the build process or at the preheating stage of the SEBM. The presence of both BCC and FCC phases in AlCrFeCoNi has also been reported in the same alloy manufactured via MA followed by SPS [198]. Jung et al. [217] synthesized AlCrFeCoMnNi HEA using laser powder bed fusion (LPBF), where its microstructure was predominantly the B2 phase (Ni-Al) and BCC solid solution (Fe-Cr); this phase constitution differed from the BCC + FCC phase constitution reported for the same alloy when using conventional melting and casting methods [218,219]. The microstructural evolution of LPBF-produced AlCrFeCoMnNi HEA has been associated with several factors, such as liquid-phase spinodal decomposition under undercooled conditions, the cubic crystal structure of the alloy, the strong crystallographic texture produced during LPBF, and the high-temperature volatility of certain alloying elements. The evaporation of volatile elements from the melting pool can give rise to compositional variations between successive deposited layers, which may subsequently alter phase transformation temperatures and the resulting phase constitution. An example is the reduction in Al content across the build, which can lead to local differences in phase stability and phase transformation. 

The influence of the fabrication route used is evident in the study conducted by Gao et al., which synthesized CoCrFeMnNi HEA and reported fine BCC grains distributed along the grain boundaries of an FCC matrix rather than being randomly distributed throughout the FCC matrix in material produced by laser 3D printing [220]. Conversely, the same nominal composition showed a single-phase FCC structure when fabricated using conventional melting and casting techniques through PM, as reported by Kucza et al. [221], Pickering et al. [222], Tsai et al. [223], and Yao et al. [224]. Another example is LBPF-produced Al_0.3_CoCrFeNi HEA, which resulted in a single-phase FCC structure, consistent with that reported for the same alloy produced through melting and casting methods [225]. Nevertheless, the rapid solidification and directional heat extraction of LBPF produced a finer columnar grain morphology, demonstrating that the fabrication route can alter grain structure, even when phase constitution remains unchanged. A similar comparison can be made for AlCoCrFeNiTi_0.5_; both laser-engineered net shaping (LENS) [226] and conventional melting and casting [227] produced ordered and disordered BCC phases, whereas MA coupled SPS resulted in a mixed BCC + FCC phase constitution [228]. Interestingly, the LENS-fabricated alloy developed a fully equiaxed grain structure rather than columnar morphology commonly associated with AM-produced alloys [229,230,231].

The effect of the fabrication route on phase formation has also been observed in AlCrCuFeNi HEA using selective laser melting (SLM)—this method produced a predominantly BCC solid solution [232] compared to a mixed BCC + FCC phase when using MA and SPS consolidation methods [233,234]. The suppression of FCC formation in this system can be attributed to the rapid cooling rates of SLM that generate significant lattice distortion and elastic strain. Collectively, these studies indicate that AM methods have significant influence on the final phase constitution and morphology of HEAs through rapid solidification, directional heat flow, and process-specific thermal histories.

## 7. Types of HEA and Their Applications

### 7.1. FCC

FCC-based HEAs are HEAs with atoms located at each corner and face center of a single cubic unit cell. They are known for their ductility and fracture toughness at low temperatures. FCC configurations include CoCrFeMnNi, CrFeCoNi, CrCoNi, and VCoNi [235]. Although FCC-based HEAs show ductility and fracture toughness, they lack strength at low temperatures. However, recent research has been exploring ways to retain their ductility and toughness while improving their strength. This is done by finding different potential strengthening mechanisms and combining or enhancing these mechanisms [236].

#### 7.1.1. TWIN/TRIP

A study conducted by Chandan [237] explored an Fe40Mn40Co10Cr10 HEA and its properties when tested at room temperature and at −100 °C, since its SFE was 37.7 (±7) mJ/m^2^ and 19.5 (±5) mJ/m^2^, respectively. This helps examine the effects of TWIN and TRIP on HEA. Here, the sample at −100 °C had a yield strength and ultimate tensile strength 1.5 times those of the samples at room temperature; additionally, the total elongation increased by almost 200% compared to the other sample. Furthermore, it was observed that there was a presence of HCP of 23%, indicating the formation of HCP due to TRIP. Additionally, the deformation indicated that the deformation was dictated by TWIP and TRIP, respectively [237].

#### 7.1.2. Solid State Strengthening

Varvenne and team [238] proposed a theory where the strengthening mechanisms of an FCC-based HEA originate from dislocation interactions along with random concentration fluctuations commonly found in HEA. This effect is known as solid solution strengthening, where dislocations migrate and glide along the {111} (110) dislocations under low Peierls stresses. 

To improve this effect, doping HEAs to produce a higher volume mismatch has led to improvements in strength. In a study by Kumar et al., doping aluminum into a CoCrFeMnNi HEA at different concentrations found that more aluminum doped into an HEA was able to increase its yield strength with minor ductility loss, as shown in Table 4 [239].

#### 7.1.3. Nanoprecipitates

As mentioned in the previous section, L12/L21-type nanoparticles are dense intermetallic phases that strengthen FCC-based HEAs [240]. A study performed by Zhao and team used (FeCoNi)_85.9_Al_8_Ti_6_B_0.1_, which was processed to form L12 nanoparticle precipitates evenly in the FCC matrix [241]. The study shows that properly aged (740 °C for 12 h) HEA formed larger L1_2_ precipitates compared to the underaged (740 °C for 20 mins) alloys, resulting in better strength as well as ductility. A similar study performed by Peng showcased an Ni_28_Co_28_Fe_21_Cr_15_Al_4_Ti_4_ HEA and the effects of L1_2_ nanoparticles and found similar results, in which longer aging times resulted in bigger precipitates. However, they also found that after 24 h of aging, its strength and ductility declined [242].

This strengthening effect is further explored in a paper by Yang, who introduced a Ni_30_Co_30_Fe_13_Cr_15_Al_6_Ti_6_ HEA that was able to perform at cryogenic temperatures of 77K, showcasing a tensile strength of 1.7 GPa and a large ductility of 51% with the help of the L1_2_ nanoprecipitates [28].

A study conducted by Teshome proposed an FCC-based HEA, CoCrFeNiMoMn, which was tested for its properties when it is as deposited and heat-treated. The X-ray diffraction results found that the heat-treated HEA formed significantly more L2_1_ precipitates that corresponded to better properties, indicated by the 42.7% and 49% increases in ultimate tensile strength [243].

#### 7.1.4. Applications

Due to FCC-HEA’s exceptional qualities at low temperatures, it has often been observed for cryogenic applications [237,244]. HEAs, such as the one formed by Chandan, showcase this by having exceptional properties at −100 °C, which indicates their potential uses in cryogenic systems, including aerospace or hydrogen storage applications [237]. Additionally, CrFeCoNiPd and FeCoNiCrMn have been researched for their potential properties for nuclear applications, as they were able to show only a 0.31% volumetric swelling at 38 DPa and a 0.10% swelling at 60 Dpa, indicating irradiation resistance [245].

### 7.2. RHEA

#### 7.2.1. Mechanical Properties of RHEA

Refractory high-entropy alloys (RHEA) are a class of HEAs that are primarily composed of refractory elements with high melting points (usually >2000 °C) such as niobium (Nb), molybdenum (Mo), tantalum (Ta), tungsten (W), vanadium (V), zirconium (Zr), hafnium (Hf), titanium (Ti), and chromium (Cr) in equiatomic or near-equiatomic ratios [246]. RHEAs normally exhibit excellent strength at high temperatures due to their severe lattice distortion and sluggish diffusion properties, but heavily inhibit the dislocation movement of the elements [247]. WMoNbTa(V) was the first RHEA that was reported by Senkov, O.N. et al. in 2010 [247]. Other mechanical properties of RHEAs also include high strength, good thermal and phase stability, and oxidation resistance. However, their mechanical performance is strongly dependent on their crystal structure and elemental composition. Additionally, they confront several difficulties that limit their practical applicability, such as significant brittleness at room temperature and limited flexibility and toughness [248]. The BCC-based HEA generally exhibited higher strength but low ductility at room temperature. However, the ductility at room temperature can be improved by substituting principal elements such as W, V, and Mo with lighter elements such as Ti, Zr, and Hf, as reported by Senkov, O.N. et al. (2011) [22]. Although certain RHEAs with Group IV principal elements demonstrate exceptionally high fracture toughness, other types of RHEAs, such as WMoNbTa(V) and WMoNbTiCr, typically show poor ductility and are unable to achieve an elongation of 10% or higher [249]. 

Additionally, a study by J. Shittu et al. (2024) investigated CrMoNbV series RHEAs and found that the single-phase BCC Cr_7_Mo_18_Nb_35_V_40_ alloy exhibited a room temperature compressive strength of approximately 1000 MPa while maintaining good plasticity [250]. Similarly, a breakthrough study by Z. An et al. (2024) showed that HfNbTiVAl_10_ alloy achieved an exceptional tensile yield strength of 1390 MPa with a plastic strain of approximately 20%, demonstrating the potential of RHEAs to overcome the conventional strength–ductility trade-off [251]. Despite their exceptional strengths, where RHEAs can exhibit ultrahigh strength (>1 GPa) at room temperature, most RHEAs generally exhibit limited tensile ductility, where tensile plasticity is often below 10% [252]. Thus, it remains a major limitation for practical applications. Surprisingly, extensive research in recent years has revealed that RHEAs demonstrate better plastic deformation under compression than tension [253]. Several other RHEA compositions have also been investigated to evaluate and improve their mechanical performance through alloy design and microstructural modification at room temperature. Selected RHEAs and their key mechanical properties at room temperature are summarized in Table 5.

RHEAs obtain their high mechanical strength from the combined operation of several strengthening mechanisms, rather than from a single microstructural feature as previously discussed under the strengthening and deformation mechanism of HEAs. The principal mechanisms include SSS, GBS, phase transformation strengthening, precipitation strengthening, and more. Meanwhile, toughening mechanisms are primarily associated with maintaining sufficient plastic deformation before fracture through dislocation activity, grain boundary cohesion, work hardening, and, in selected metastable systems, transformation- or twinning-assisted deformation. This combination is particularly important for RHEAs because their BCC structures not only provide excellent strength and thermal stability but can also result in limited room-temperature ductility and premature brittle and intergranular fractures. Table 6 highlights the fundamental principles of each mechanism to be discussed, along with the appropriate applications and examples: 

#### 7.2.2. Physical and Chemical Properties of RHEA

High-temperature oxidation resistance is a vital property of RHEAs, particularly for applications in extreme environments. When exposed to high-temperature oxidizing environments, RHEAs will undergo oxidation, which results in the formation of oxide layers on their surfaces. The density and stability of these oxide films can potentially provide excellent oxidation protection. In particular, studies found that elements such as Cr and Al can help contribute to the formation of dense and stable protective oxides, which include Cr_2_O_3_ and Al_2_O_3_ films by limiting the diffusion of oxygen into the underlying alloy [262]. However, the oxidation resistance of RHEAs can deteriorate when exposed to extreme temperatures that exceed 1000 °C due to thermal stresses, which lead to the rapid oxidation of RHEAs [262]. Oxidation behavior is also highly dependent on the specific alloying elements present in RHEAs. There are several studies that have investigated high-temperature oxidation behavior of RHEAs with different alloying compositions to improve their oxidation resistance. Rational alloy design and careful selection of the alloying elements are extremely essential for developing RHEAs with enhanced oxidation resistance for high-temperature applications. The findings are summarized in Table 7.

Corrosion resistance is also another important key property of RHEAs, particularly for applications that are exposed to acidic, alkaline, saline, and other corrosive environments. Studies have demonstrated that several RHEAs exhibit superior electrochemical corrosion resistance, with some RHEAs achieving corrosion current densities as low as 10−7 A/cm^2^ in acidic, neutral, and alkaline environments [267]. This superior corrosion resistance is mainly attributed to their multi-elemental composition and the formation of stable passive films on the alloy surface, specifically refractory elements such as Cr, Mo, and Nb, which contribute to the formation of dense and protective oxide films enriched with Cr, Mo, and Nb oxides. These passive films act as a protective barrier against the penetration of aggressive corrosive media such as chloride ions [268]. Apart from these attributes, studies by S. Pal et al. (2023) also investigated the corrosion behavior of AlCoCrFeMo in 3.5% NaCl solution and found that the addition of W and V can promote the formation of protective passive films, which significantly lowers the corrosion rate compared to traditionally used nickel- or steel-based alloys [269]. Another study by Z.H. Han et al. (2024) investigated the electrochemical corrosion behavior of VNbTa by immersing it in sulfuric acid and sodium chloride solutions. They found that VNbTa exhibits outstanding corrosion resistance compared with the commonly used stainless steel 316, which forms dense oxide layers of Ta_2_O_5_ and Nb_2_O_5_ on the protective passive film [270]. Moreover, H.C. Ozdemir et al. (2023) also studied the electrochemical corrosion behavior of Hf_27_Nb_12_Ta_10_Ti_23_Zr_28_ alloys in different highly acidic and alkaline solutions and reported that they exhibit corrosion resistance that is far superior compared to the conventional CoCrMo biomedical alloy [271]. Therefore, these findings demonstrate the importance of the addition of refractory elements in controlling the formation and stability of the protective passive films that help to promote better corrosion resistance of RHEAs. 

#### 7.2.3. Irradiation Resistance

Furthermore, irradiation resistance is also another key property that is important for RHEAs, particularly for applications that are heavily involved in nuclear reactors and components that are exposed to high-energy radiation environments. Long-term exposure to irradiation can induce various forms of material degradation, including lattice defects, dislocation loops, helium bubble formation, swelling, and embrittlement, which compromise the mechanical integrity and service life of structural materials [272,273]. Recently, RHEAs have attracted attention for such applications due to their complex multi-elemental composition and severe lattice distortion properties, which are being “watch listed” as a potential mitigation strategy that can provide multiple defect-trapping sites and suppress the growth of irradiation-induced defects, thereby improving the microstructural stability of RHEAs under irradiation [253].

Several RHEAs have been investigated throughout the years to evaluate their resistance to irradiation-induced damage. Selected RHEAs investigated for irradiation resistance and their key findings are summarized in Table 8 below.

#### 7.2.4. Doping of Non-Metallic Interstitial Elements

Furthermore, the addition of non-metallic interstitial elements, which include carbon (C), nitrogen (N), oxygen (O), and boron (B), is also an alternative effective approach for improving the mechanical properties of RHEAs, where they will occupy the interstitial sites within the RHEA lattice. Due to their relatively small atomic sizes, these elements introduce additional lattice distortion and local compositional fluctuations, which will hinder the movement of dislocations [277]. As a result, resistance to plastic deformation increases, resulting in enhanced solid solution strengthening. For instance, doping at 1.6 at.% of interstitial elements, such as O and N, in TiVNbTa was reported by Lee et al. (2021) to increase its yield strength by approximately 300 MPa [278]. However, the effect of interstitial doping on RHEA depends strongly on the type and concentration of the dopant. In other words, an excessive amount of interstitial elements may lead to the formation of secondary phases, such as oxides, nitrides, or other precipitates, which may increase brittleness and ductility [279]. Thus, the solubility of the interstitial elements within the RHEA matrix, and their interactions with the constituent elements, must be considered. An appropriate concentration of interstitial elements could enhance the strength of a specific RHEA while maintaining a tolerable level of ductility, making interstitial doping a promising strategy for overcoming the strength–ductility trade-off in RHEA. 

#### 7.2.5. Applications

With RHEA’s unique mechanical and physicochemical properties, RHEAs are a promising material, especially for aerospace, nuclear energy, gas turbines, petrochemicals, and many more, particularly components that are exposed to extreme temperatures and mechanical stresses.

In the aerospace field, RHEAs have emerged as a potential replacement for traditional nickel-based superalloys due to their excellent oxidation resistance and ability to retain mechanical strength at extreme temperatures. These properties make RHEAs promising candidates for high-temperature structural applications, such as gas turbine blades, aircraft engine components, rocket engines, and spacecraft [280]. Specifically, RHEAs have the potential to be used in a critical thermal protection component of spacecraft, such as the nose cones and wing leading edges, where they can help to maintain structural integrity, functionality, and safety under extreme thermal conditions. Their excellent high-temperature strength and thermal stability enable them to withstand intense heat and protect the spacecraft from overheating and thermal damage. Based on research by Long X et al. (2023), the HfNbTiZr alloy exhibited superior long-term strength, fracture toughness, and oxidation resistance at temperatures above 1200 °C compared to the traditionally used nickel-based superalloys [281]. 

In addition, RHEAs also show a significant potential for gas turbine applications, particularly in turbine blades and combustion chambers that operate under extreme and harsh conditions. Their excellent high-temperature performance allows them to maintain mechanical strength and structural stability in these demanding environments. Consequently, the use of RHEA could enable gas turbines to operate at higher temperatures, thereby improving power generation efficiency and helping to address increasing global energy demands [253]. 

Aside from the above-mentioned, RHEAs are also promising candidates for critical components of nuclear reactors, such as cladding, vessels, and pipelines, where material performance is crucial for ensuring reactor safety and economic operation [253]. RHEAs exhibit not only excellent high-temperature stability but also remarkable resistance to irradiation, which is essential for maintaining material integrity under prolonged exposure to radiation. Among the various RHEAs investigated for nuclear applications, WTaCrV RHEA has received considerable attention. D. Kalita et al. reported that WTaCrV RHEA demonstrated excellent microstructural stability under a high dose of He ion irradiation, which indicates its potential for use in critical nuclear reactor components [282]. In addition, RHEA coatings such as CrMoNbTaW have also been investigated as corrosion-resistant functional coatings for Gen-IV nuclear systems, particularly molten salt reactors (MSRs). According to a recent report by the OECD Nuclear Energy Agency, CrMoNbTaW coatings exhibit outstanding corrosion resistance and can form a protective layer on stainless steel surfaces under harsh operating conditions, including high-temperature environments [283]. Thus, producing RHEA-based coatings could provide a promising approach to mitigating corrosion-related issues in MSRs and enhancing the long-term safety and operational reliability of nuclear reactors. 

Furthermore, RHEAs are also promising candidates for applications in the petrochemical industry, where materials are often exposed to harsh operating conditions, including extreme temperatures, high pressures, and corrosive environments. There are several key components in petrochemical facilities, such as cracking furnace tubes and reformer tubes, operating continuously under high temperature, high pressure, and sulfur-containing environments. Consequently, these components require materials with excellent high-temperature strength, oxidation resistance, and resistance to sulfur-induced corrosion [253]. As discussed earlier, conventional materials such as nickel-based superalloys and heat-resistant steels may experience degradation in mechanical strength and corrosion resistance at temperatures above 900 °C, whereas a RHEA-based material can maintain excellent mechanical strength and structural stability at temperatures exceeding 1200 °C while also demonstrating good oxidation and sulfur corrosion resistance [253]. Therefore, these applications of RHEA in critical petrochemical components have the potential to improve the safety, stability, and service life of petrochemical facilities. 

### 7.3. Nanostructured HEAs

Nanostructured or nanocrystalline (NC) materials are nanomaterials with nano-sized grains that induce different mechanical and functional properties. For NC HEAs, most have been made using transition metals, which are separated into aluminum-containing or non-Al-containing HEAs. These types of NC HEAs are commonly made using metal alloying or high-pressure torsion (HPT) [284].

**Figure 3:**
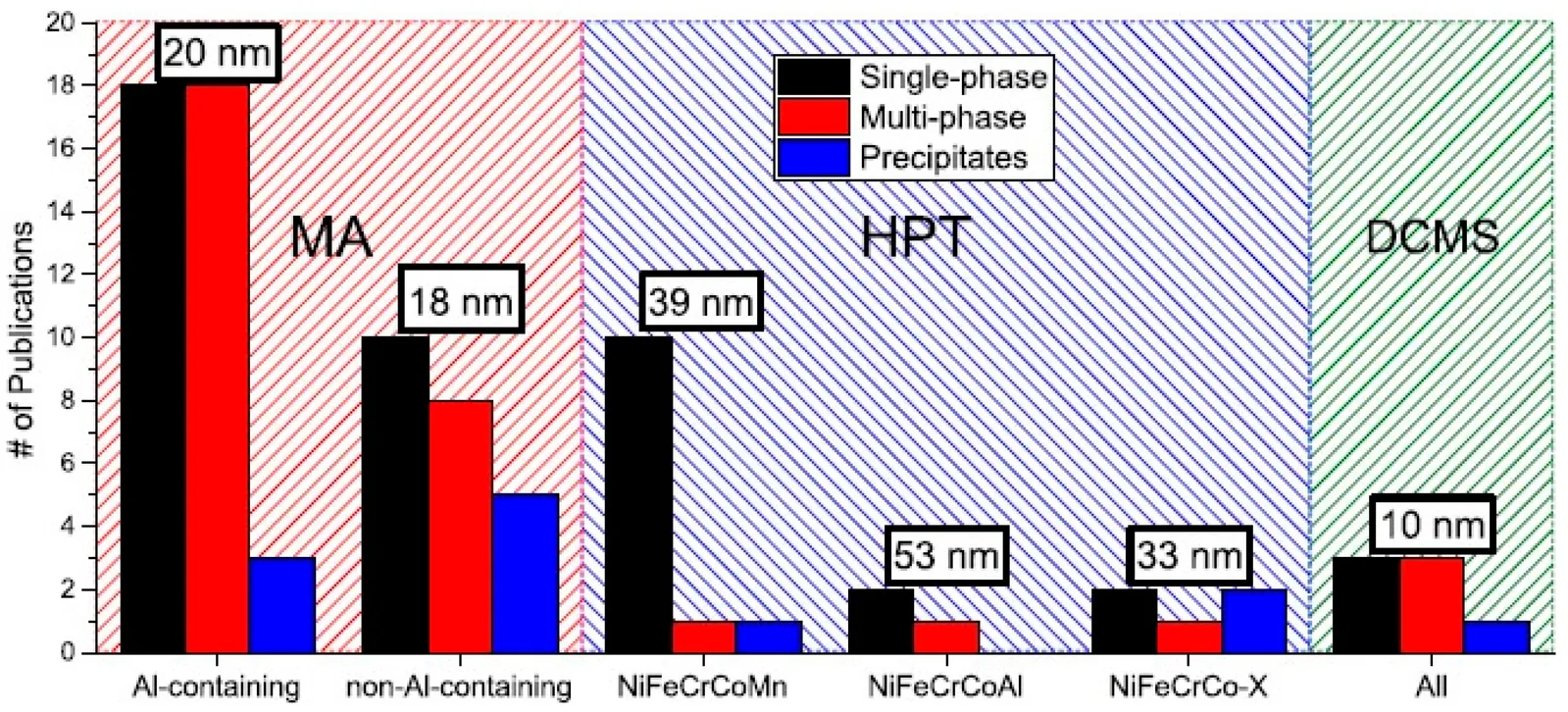
Phase formation tendencies in transition NC HEAs from metal alloying or high-pressure torsion (HPT) and DC magnetron sputtering (DCMS). Reprinted from Ref. [284].

These processes require significant plastic deformation, which requires more processing time, and as seen in Figure 3, they exhibit a larger grain size compared to DC magnetron sputtering (DCMS). This effect is more noticeable in single-phase and multiphase HEAs. However, DCMS-processed HEAs are only able to produce thin films of up to 5 µm, which makes it difficult to determine properties [284].

Non-transitional metal HEAs have no significant tendency within each processing method. Other than that, interstitial HEAs have been widely used due to their single-state hardening due to the addition of nitrogen and carbon that forms nano-sized precipitates or multiphase structures, which have proven to have great mechanical properties [284].

#### 7.3.1. Mechanical and Thermal Properties

**Figure 4:**
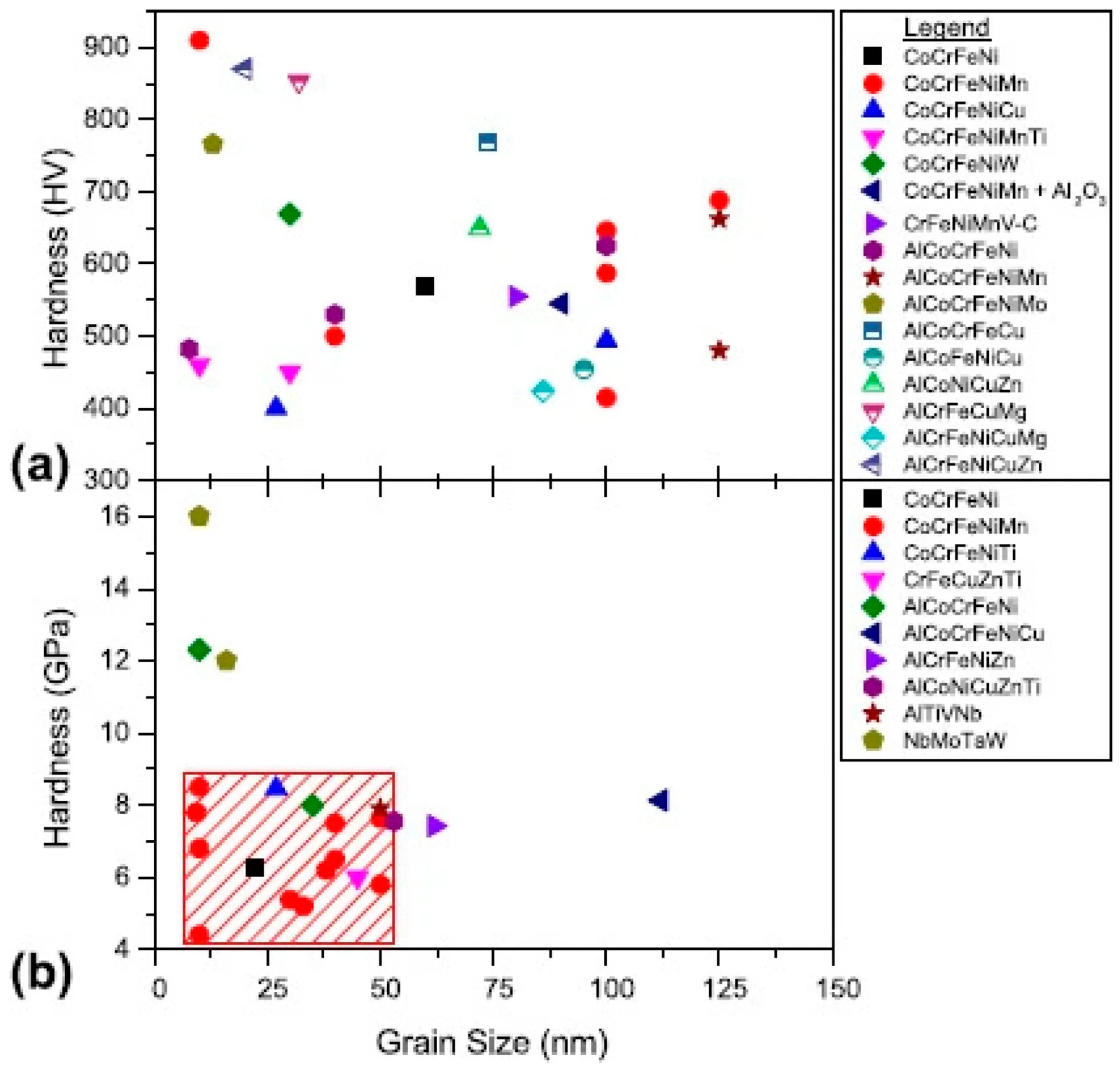
Different NC HEAs’ (**a**) Vickers micro-hardness (HV) and (**b**) hardness measured in GPa. Reprinted from Ref. [284].

The properties of NC HEAs have been of note, as they are able to outperform conventional alloys in hardness and tensile strength when compared, with values from 400 to 700 HV, and up to 16 GPa, respectively (Figure 4) [284]. This can be observed in the figure below. Additionally, refractory HEAs were able to achieve even higher hardness values of 64 and 70 GPa [285,286].

**Figure 5:**
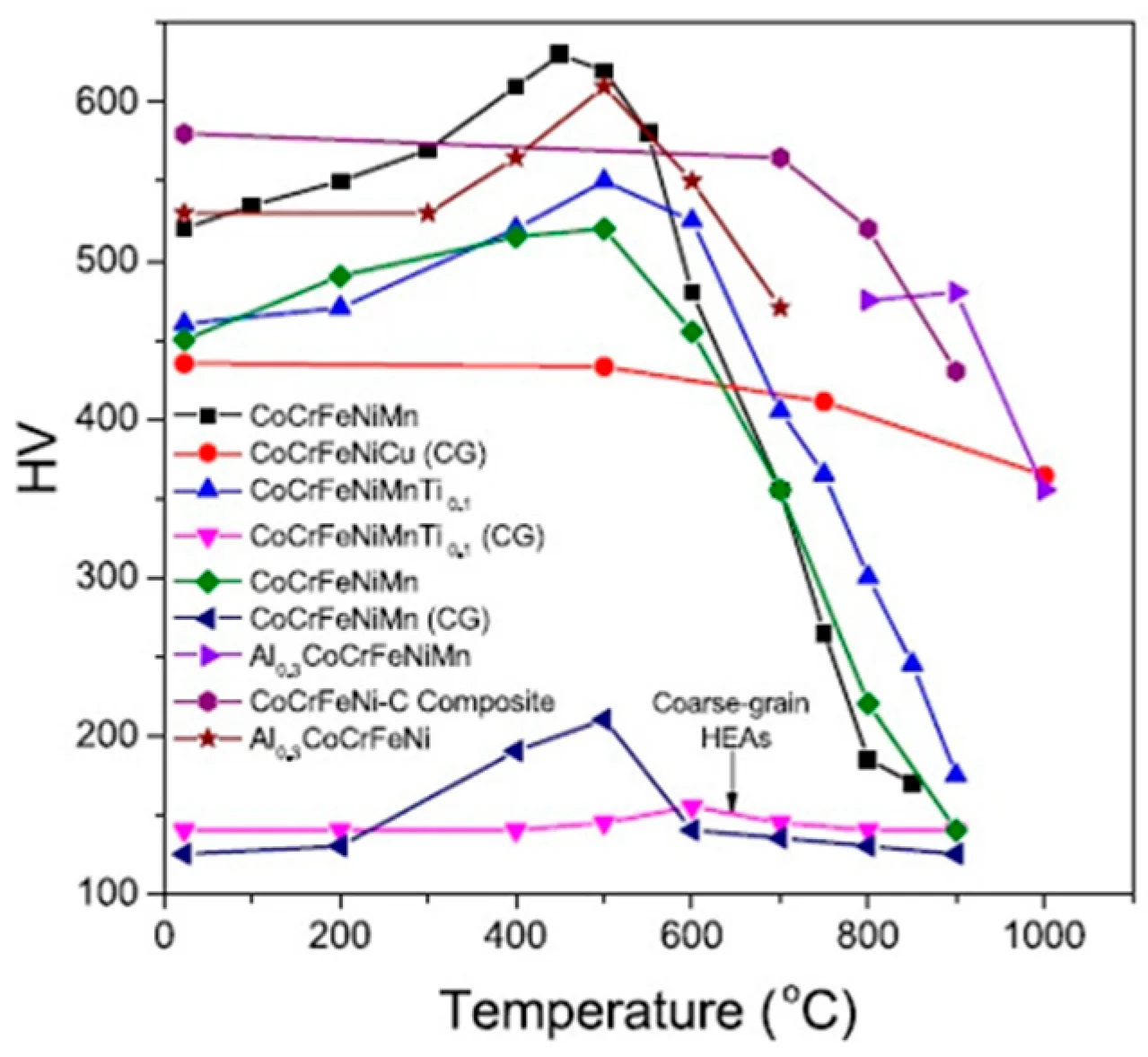
Hardness values of various HEAs of coarse-grained (CG) and NC HEAs. Reprinted from Ref. [284].

When looking at HEAs’ thermal stability, it is observed that the HEA with a nanograined structure performed better than those with coarse grains, experiencing a smaller drop in hardness when brought up to temperatures of 600 °C to 1000 °C, as seen in Figure 5. A study performed by Shahmir on CoCrFeNiMnTi_2_ and CoCrFeNiMn found that they both increase in hardness up to 600 °C, reaching 450 HV before dropping to lower hardness values above that temperature, as grain growth begins only after 600 °C. As seen in Figure 5, most other HEAs follow this trend [287].

#### 7.3.2. Functional Properties

NC HEAs also have various other functional properties such as corrosion resistance, electrochemical, magnetic, and electrical properties. These properties, however, only exist in very specific types of NC HEAs, most commonly Al-containing HEAs; thus, only a few studies regarding their functional properties have been published [287]. 

NC HEAs have been widely observed for their thermoelectric properties due to their core effects, which affect electron movement and reduce thermal conductivity. Thus, NC HEAs’ Seebeck coefficient is widely observed to investigate this property since it measures the amount of voltage produced when a temperature difference is present [284,288].

**Figure 6:**
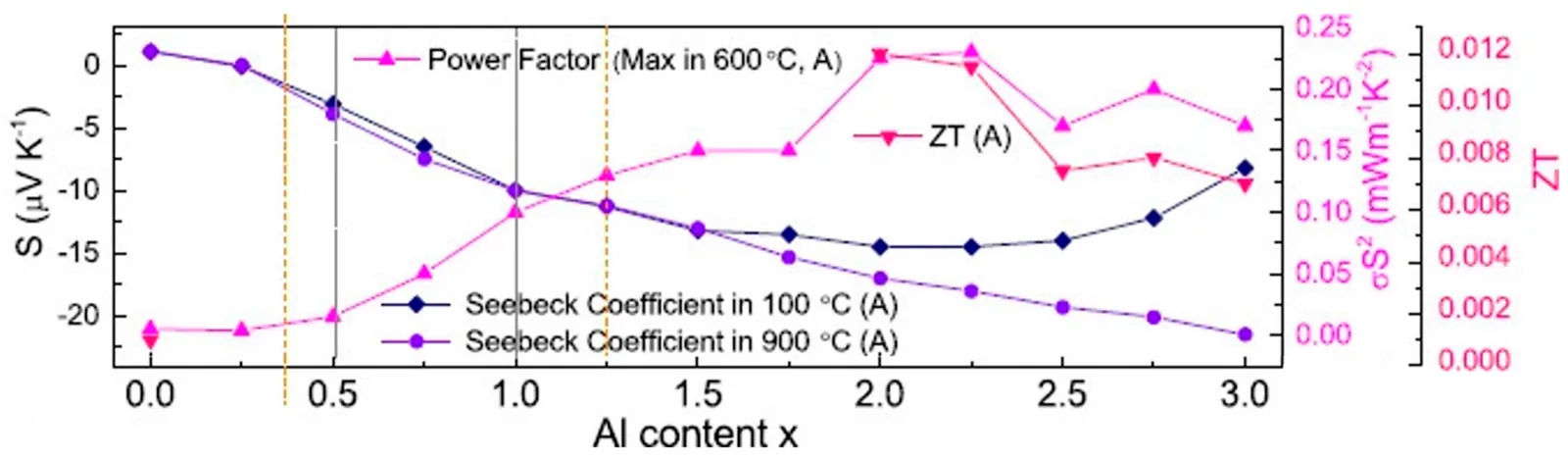
Seebeck coefficient of Al-containing HEA with various Al contents. Reprinted from Ref. [284].

A study performed by Shi investigated AlxCoCrFeNi and found that an increase in Al affected both electrical and thermal conductivity. However, it was found that the structure of the HEA was affected more, since when its symmetry and homogeneity were increased, its thermal conductivity and Seebeck coefficient increased, as seen in Figure 6 [284].

The magnetic properties of HEAs largely depend on the material used in the HEA; for example, Cr reduces the magnetic properties since it is antiferromagnetic, and Cu reduces magnetic properties given that it is diamagnetic [284].

Next, NC HEAs have low electrical conductivity due to the scattering of electrons as their grains are nanosized. It was also found that Al-containing HEAs with structures that are more homogenized have lower electrical resistance, while plastically deformed HEAs have higher electrical resistance [289]. A study performed by A. Rohr found a (TaNb)_1−*x*_(ZrHfTi)*_x_*, which has a BCC nanostructure with both crystalline and amorphous features, with potential applications as a superconductor [290].

Corrosion has no significant relationship with its grain size but is generally dictated by the density of grain boundaries, where a higher density will significantly lower the activation energy of corrosion [284]. Additionally, the addition of certain elements, such as copper and aluminum, has been shown to decrease corrosion resistance since they form BCC precipitates inside the FCC matrix, turning into a galvanic cell, thus promoting corrosion [21,291]. 

To reduce these effects, heat treatment can be performed to allow the BCC precipitates to transform into the FCC matrix [21]. Other stable metals have been added to HEA to observe the effects on its corrosion resistance, such as Cr being added to AlCuFeNi, which was able to reduce the corrosion rate [21].

However, when elements like Mo are added, they can show different properties since they are able to resist Cl^−^ anions, while when added with Ti in a TiZr_0.5_NbCr_0.5_V*_x_*Mo*_y_* HEA, corrosion resistance to NaCl decreases, whereas resistance to H_2_SO_4_ increases [291,292].

#### 7.3.3. Electromagnetic Shielding

HEAs have been observed for electromagnetic shielding purposes due to the use of conductive metals within a HEA, which is able to have high electrical conductivity, allowing for good electromagnetic absorption. Materials such as Cu and Mn are often optimized for this purpose, and it was found that HEAs were able to perform better compared to other materials, as reviewed by Zhang [284].

In a study performed by Zhang, electrostatic spinning was used to form FeCoNiCuMn HEA into a porous carbon nanofiber, obtaining a minimum reflection loss of −65.8 dB and a maximum absorption bandwidth of 7.68 GHz [293].

#### 7.3.4. Gas Sensor

HEAs can have many active sites that interact with gas with great selectivity and sensitivity; this has led to HEAs being used in gas sensors for environmental monitoring. A study performed by Naganaboina used Gd_0.2_La_0.2_Ce_0.2_Hf_0.2_Zr_0.2_O_2_ as a high-entropy oxide-based chemiresistor to detect methane. The compound was able to show high selectivity to methane with an experimental detection limit of 25 ppm, along with lower energy demands of 50 nW of power. This can be used in industrial environments as a precaution against accidents.

Another report using high-entropy metal oxide (Cu_0.2_Ni_0.2_Zn_0.2_Cd_0.2_Co_0.2_) WO_4_ was able to measure ammonia and triethylamine. It was reported to be able to detect both compounds at higher temperatures, up to a tested 400 °C, and had a low limit of detection of 0.01 ppm with great repeatability [294]. 

A novel HEA, AuAgCuPdPt HEA-modified MoS_2_ nanosheet, was able to improve hydrogen adsorption at 80 °C due to the combination of strong and weak hydrogen adsorption capabilities [295].

#### 7.3.5. Biomedical Applications

Nanocatalysts have attracted interest, citing their cost-effectiveness and resilience in harsh conditions; however, they have some limitations in their catalytic efficiency; thus, HEAs are emerging as a potential solution to assist in these problems [296].

A paper by Ai explores ultra-small high-entropy alloy nanoparticles (US-HEANPs) by fabricating PtPdRuRhIr nanoparticles that can have peroxidase-like activity, which catalyzes hydrogen peroxide into toxic hydroxyl radicals that were able to treat tumors. When tested at an HEA concentration of 100 µg mL^−1^, cancer cells were reduced by 75%, while other cells had a 90% survival rate, indicating high selectivity in tumor targeting and biocompatibility [297].

Other applications, such as bone substitutes using elements such as Ti, Zr, Mn, Al, Nb, and Fe, are often seen as potential substitutes. A review conducted by Lv found various potential uses for oral use, bone repair materials, repair scaffolds, and biocompatible coatings. However, more research still needs to be performed, as most of the research done to date still lacks long-term data but has proven well as a proof-of-concept [298].

#### 7.3.6. Energy Storage

Due to HEA’s ability to be a multicomponent oxide, it has the potential to perform much better than existing supercapacitors. A study conducted by Ma used Prussian blue analogs (PBAs), which are efficient sodium storage materials, and made a high-entropy PBA consisting of Fe, Mn, Ni, Cu, and Co. The new capacitor was able to have a Coulombic efficiency of nearly 100% after more than 3000 cycles, with a 68 mAh g^−1^ specific discharge capacity [299].

Another HEA, such as FeNiCoMnMg, was electrospun on a carbon nanofiber and achieved a specific energy density of 203 F g^−1^ and 21.7 Wh kg^−1^ [300]. Additionally, a high-entropy NiCoFeZnSb was able to maintain 82% efficiency after 10,000 cycles, with a capacitance of 1850.0 C g^−1^ at 1 A g^−1^ [301]. Other similar high-entropy materials were also able to give promising results, such as high-entropy hydroxides, high-entropy oxides, and high-entropy MXenes, as reported by Ren [302].

#### 7.3.7. Hydrogen Storage

NC HEAs have been studied for their potential use in hydrogen storage due to their core effects, especially lattice distortion, making them highly suitable. A study conducted by Chen tested its capabilities in an HEA consisting of TiZrFeMnCrVx, with varying amounts of vanadium. Chen found that the higher content of vanadium was able to absorb more hydrogen, while all the alloys were able to absorb 90% of hydrogen within 76 s at 30 °C. However, the vanadium at 1% had the best reversibility with a capacity of 98% after 15 cycles [303]. Other materials such as Mo were tested in a TiZrHfMo_x_Nb_2−x_ (*x* = 0, 1, 2) HEA, where an increasing amount of Mo compared to Nb enables more hydrogen to migrate within the lattice [304].

### 7.4. Eutectic High-Entropy Alloys (EHEAs)

First, conventional single-phase HEAs face a practical casting limitation. This is because they contain multiple elements in high concentrations, which causes them to solidify over a wide range of temperatures. This leads to poor liquidity, poor castability, and considerable compositional segregation that restricts large-scale production [30].

**Figure 7:**
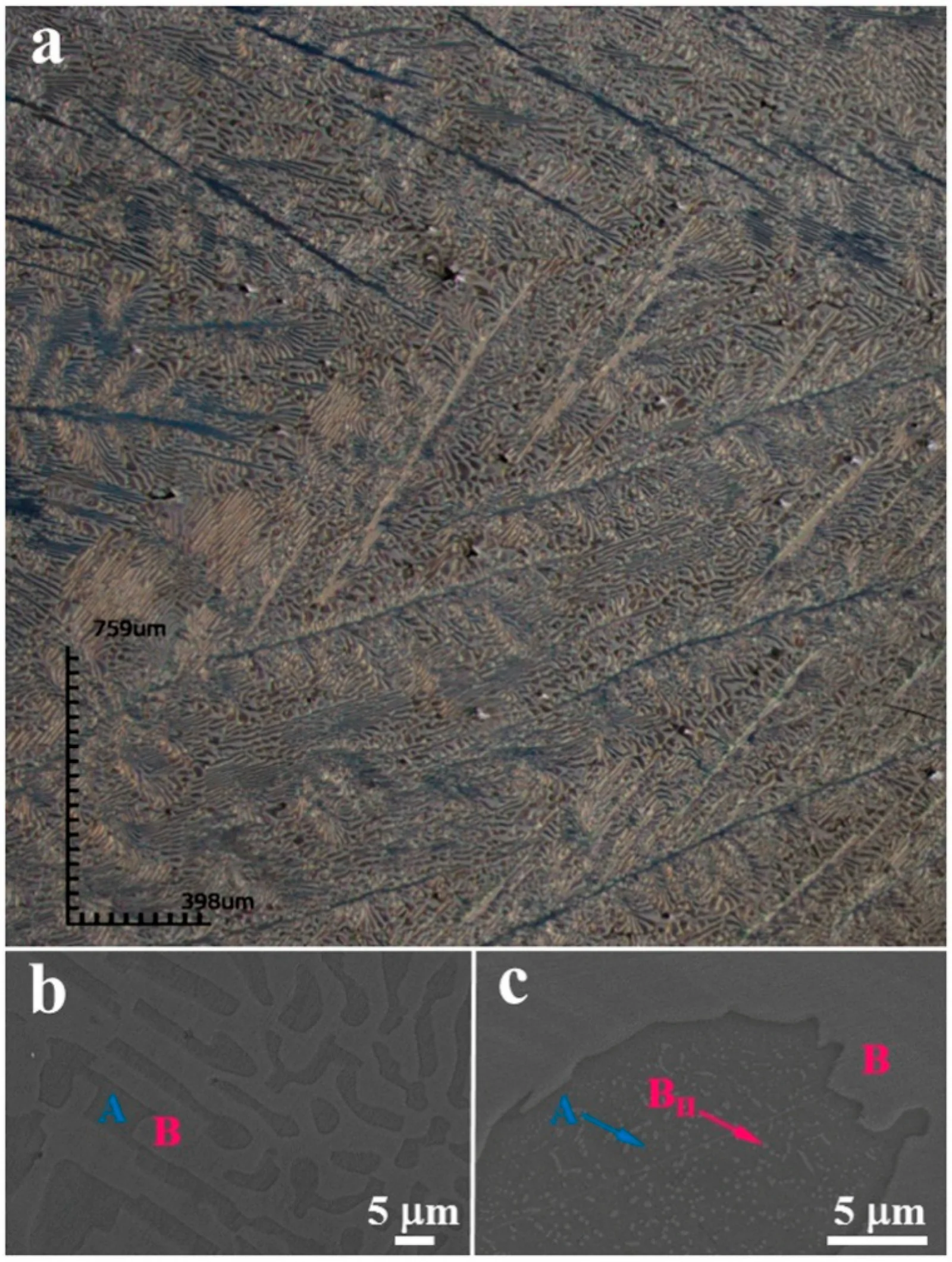
(**a**) LSCM image of the eutectic microstructure in AlCoCrFeNi_2.1_; (**b**) SEM view of the lamellar dual-phase structure; (**c**) magnified view showing precipitates within one phase, with A and B marking the two compositionally distinct phases. Reprinted from Ref. [30].

EHEAs address this problem through the eutectic reaction. The result is a specific alloy composition that directly solidifies from the liquid into two solid phases at a single fixed temperature simultaneously. This effectively eliminates the wide freezing range and the segregation that it causes. The EHEA AlCoCrFeNi_2.1_ is engineered at the eutectic point to solidify into an alternating lamellar structure of soft, ductile FCC and hard, strong B2 (ordered BCC) phases (Figure 7) [30].

#### 7.4.1. Eutectic Solidification and Dual-Phase Microstructure

The as-cast AlCoCrFeNi_2.1_ EHEA manages to achieve a tensile strength of 944 MPa with a fracture elongation of 25.6% straight out of casting, with no post-processing needed [305]. This performance comes from partitioning strength and ductility into separate phases rather than relying on a single mechanism. The soft FCC lamellae would absorb any strain, while the hard B2 lamellae would resist deformation, which gives it a naturally self-organized composite. A secondary strengthening layer also forms inside the coarse lamellae, including nanocrystalline BCC precipitates that nucleate within the B2 phase, and L1_2_ precipitates within the FCC phase, both coherently with their respective matrices [305].

Building upon this base composition, Zhang et al. developed a “steel-frame” variant of the eutectic microstructure by combining FCC, L1_2_, and B2 phases, which hinders dislocation motion and delays any crack propagation. Since the load-bearing framework redistributes stress around defects, the alloy showed almost no notch sensitivity. The tensile properties of notched specimens were comparable to, and could even exceed, un-notched samples from across four different notch radii [306].

#### 7.4.2. Microstructural Refinement via Processing

**Figure 8:**
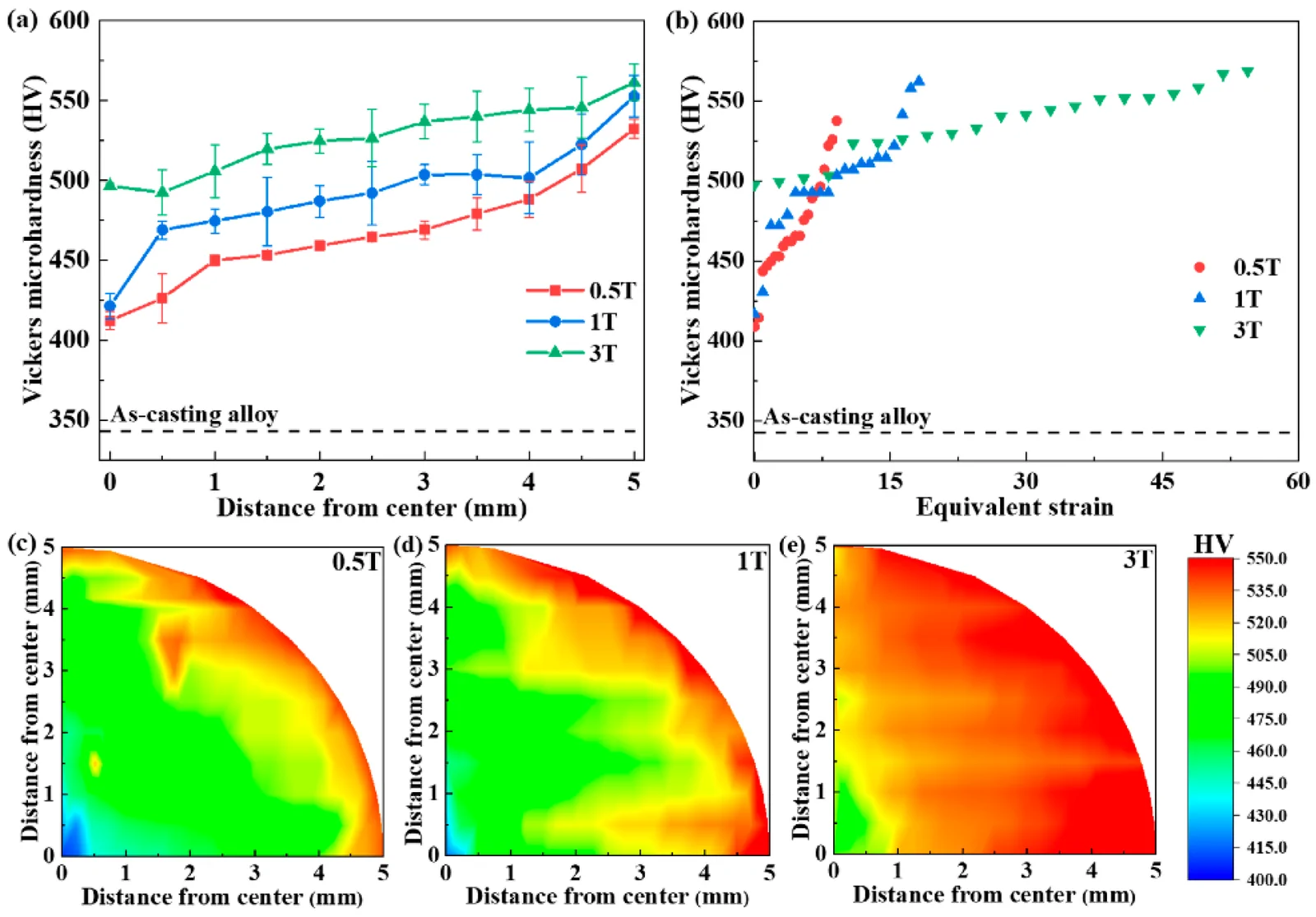
Vickers microhardness of AlCoCrFeNi_2.1_ EHEA as a function of (**a**) distance from center and (**b**) equivalent strain, with (**c**–**e**) corresponding hardness contour maps, after high-pressure torsion at 0.5, 1, and 3 turns. Reprinted from Ref. [307].

Severe plastic deformation was found to further amplify the strength of EHEA. Wang and colleagues subjected AlCoCrFeNi_2.1_ to high-pressure torsion (HPT) at 6 GPa, which refined the coarse eutectic lamellae into an ultrafine-grained structure. They found that microhardness rose to approximately 575 HV after three turns, and the ultimate tensile strength nearly doubled from around 1100 MPa in the as-cast state to approximately 1900 MPa post-HPT [307]. This essentially illustrated that the eutectic microstructure is not just an as-cast convenience, but it is also a strong starting point for further thermomechanical strengthening, as depicted in Figure 8 [307]. 

#### 7.4.3. Applications

Considering the above-mentioned, the primary appeal of EHEA lies in its castability. Since the eutectic composition solidifies at a single fixed point rather than over a wide range, near-net-shape castings can be produced directly with a strong strength–ductility combination without the typical expensive homogenization treatments that single-phase HEAs typically need [30,305]. This increases the attractiveness of EHEA for structural components manufactured through conventional or additive casting routes.

Furthermore, EHEA is also an emerging candidate for nuclear structural applications. Malinker and colleagues investigated the ion irradiation resilience of AlCoCrFeNi_2.1_ and found that the dual-phase lamellar microstructure managed to retain a strong structural integrity, even under Ne-ion irradiation up to 1.5 displacements per atom, with the L1_2_ phase showing some dose-dependent disordering while the B2 phase retained its order [308]. This combination of thermal stability, mechanical performance, and irradiation resistance positions EHEAs as promising candidates for internal reactor components and other structural components that are exposed to radiation environments.

### 7.5. High-Entropy Superalloys (HESAs)

High-entropy superalloys (HESAs) apply the same γ/γ′ architecture, but with one difference: they replace the single-element nickel matrix with a multi-principal-element, high-entropy FCC matrix. This design criterion necessitates a high configurational entropy of mixing (ΔS_mix_ > 1.5R) alongside the FCC γ matrix and L1_2_ γ′ precipitate [309]. This concept was first demonstrated by Yeh, Tsao, and colleagues, who developed non-equimolar Al-Co-Cr-Fe-Ni-Ti alloy systems containing a stable FCC γ matrix with a uniform dispersion of L1_2_ γ′ particles, managing to achieve densities below 8.0 g/cm^3^ at a lower raw material cost than conventional Ni-based superalloys [309].

#### 7.5.1. Γ/γ′ Microstructure and Precipitation Strengthening

The strengthening mechanism builds directly upon the precipitation strengthening principles covered in Section 4.4. Sluggish diffusion slows precipitate coarsening far more effectively than in a conventional nickel matrix, which allows fine γ′ dispersions to remain stable for longer at elevated temperatures.

**Figure 9:**
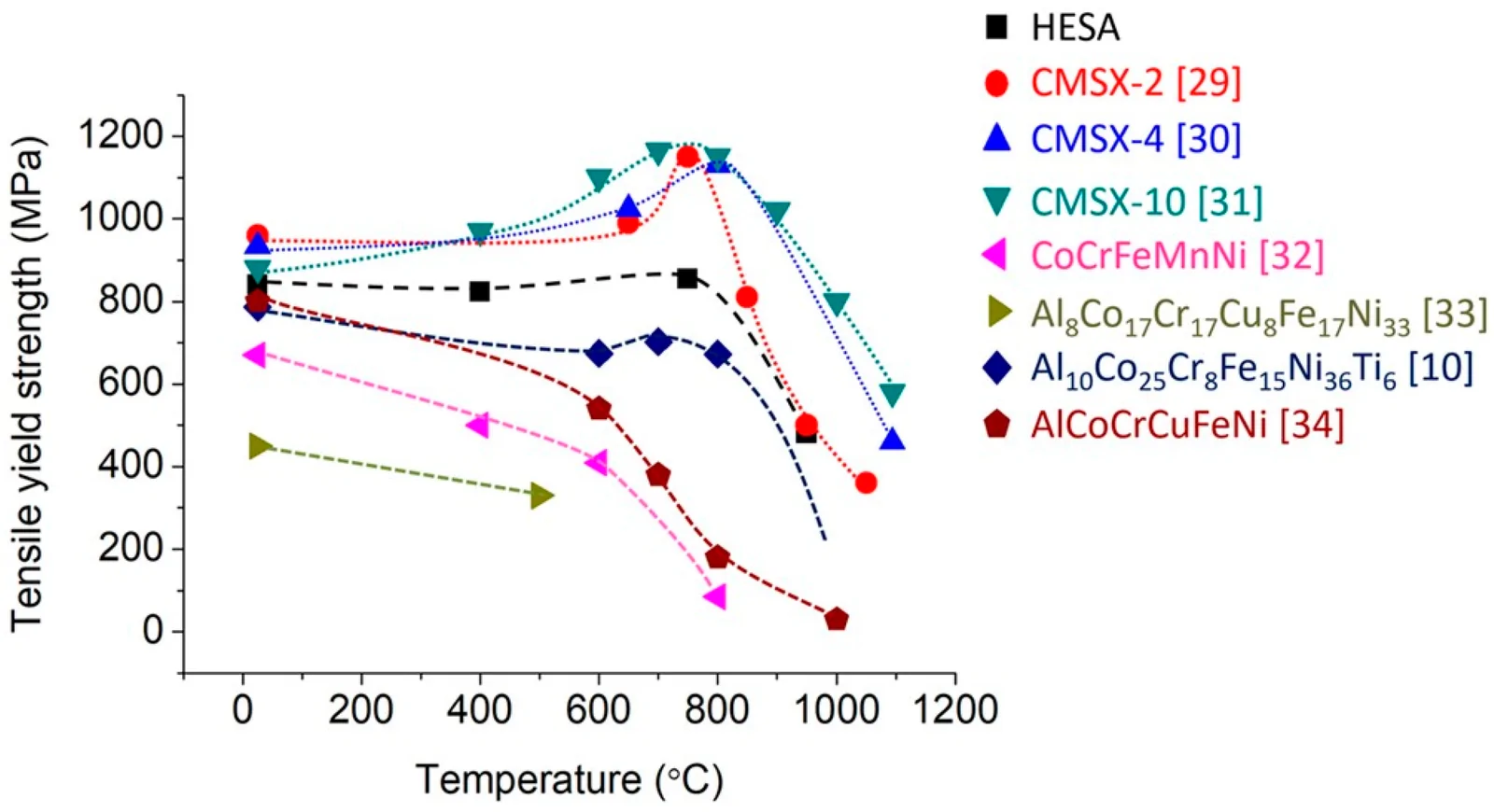
Tensile yield strength versus temperature for HESAs compared against Ni-based superalloys and other reported high-entropy alloys, from room temperature to elevated temperature. Reprinted from Ref. [310].

Tsao et al. characterized a Ni_47.9_Al_10.2_Co_16.9_Cr_7.4_Fe_8.9_Ti_5.8_Mo_0.9_Nb_1.2_W_0.4_C_0.4_ HESA and found that its tensile yield strength actually surpassed other reported HEAs from room temperature through to an elevated temperature, with a creep resistance of 982 °C, comparable to established Ni-based superalloys (Figure 9) [310]. Under creep loading, the γ/γ′ interface developed a parallel raft microstructure, resulting in superior creep resistance also seen in conventional superalloys [310].

Moreover, recent work has pushed the performance of HESAs further. Gao and colleagues have developed a Ni-Co-Cr-Al-based HESA that was strengthened by coherent, stable nanoscale L1_2_ precipitates in a low-stacking-fault-energy FCC matrix. It had a room-temperature yield strength of 1167 MPa, ultimate tensile strength of 1632 MPa, and 14.8% elongation, with the UTS remaining around 1100 MPa at 750 °C, exceeding both traditional FCC-type HEAs and typical wrought Ni-based superalloys at that temperature [311]. These exceptional properties were attributed to multiple concurrent work-hardening mechanisms, namely stacking faults, deformation-induced microbands, dislocation cells, and dislocation shearing of the γ′ precipitates via the antiphase boundary mechanism [311].

#### 7.5.2. Thermal Stability and High-Temperature Performance

The combination of sluggish diffusion in the matrix and coherent γ′ precipitation gives HESAs thermal stability that can extend beyond the operating envelope of conventional superalloys. These HESAs have the potential to function at temperatures beyond 1000 °C, a region where nickel-based superalloys begin to suffer oxidation and phase instability [88]. This is a rather significant claim given how mature and heavily optimized nickel superalloy metallurgy already is. 

HESAs are still considered early-stage technology. The γ′ solvus temperature (the temperature at which the strengthening precipitates dissolve back into the matrix) in reported HESAs is still generally lower than that of mature commercial superalloys, and phase instability from competing intermetallic phases such as B2 and L2_1_ still remains a design challenge that current alloy development is actively working on to resolve [310].

#### 7.5.3. Applications

Nickel-based superalloys, despite their maturity, are heavily limited by high density, comparatively low creep strength at extreme temperatures, poor thermal conductivity, difficulty machining, and low fatigue resistance. All of these are driving the search for more viable alternatives [312]. With that being said, HESA is positioned as a direct candidate to fill this gap, fitting naturally into components such as turbine blades, aircraft engine hot-section components, and gas turbines since HESA has the potential to match or exceed the creep and tensile performance of conventional superalloys at lower densities and lower raw material costs [309,312]. Beyond aerospace, the same high-temperature stability and oxidation resistance make HESAs relevant to power generation turbines, where efficiency gains scale directly with how hot a turbine can safely run [312].

## 8. Conclusions

HEAs have emerged as a versatile class of advanced engineering materials with the potential to provide a fundamentally unique approach to alloy design via the utilization of multiple principal elements with equimolar or near-equimolar properties. This review has presented the distinctive behavior of HEAs, driven by the combined effects of configurational entropy, composition, thermodynamic stability, atomic interactions, microstructural development, and processing pathways. All factors established collectively facilitate a broader design space, ensuring that the mechanical, thermal, chemical, and functional properties of HEAs can be tailored to meet specific engineering requirements [1,313].

The phase formation and stability of HEAs provide a predictive model of a HEA phase. However, the individual limitations have demonstrated that no single criterion has the ability to universally determine the stability of a HEA system. Consequently, phase prediction requires the consideration of both thermodynamic and kinetic factors concurrently, along with composition and processing conditions. The fabrication route of a HEA could produce varying phase assemblages and microstructure, even for similar nominal compositions; hence, the delicate consideration of every factor during phase prediction is extremely significant [313]. 

The strengthening and deformation behavior of HEAs further demonstrates the importance of microstructure control. SSS and GBS provide the fundamental contributions to the strength of HEAs, while phase transformation and precipitation strengthening mechanisms contribute to additional pathways for controlling the balance between mechanical strength and the ductility of HEAs. The ability of both TRIP and TWIP mechanisms to generate deformation-induced barriers, along with the strengthening contribution of nanoscale precipitates, has successfully demonstrated the potential for HEAs to overcome the strength–ductility trade-off faced by conventional materials. Nonetheless, the effectiveness of these mechanisms discussed in the review remains dependent on the composition of the alloys, together with phase stability, grain structure, precipitate characteristics, and deformation conditions rather than on any individual strengthening mechanism [92,238,314]. 

Fabrication represents an integral component of HEA design, as the review has demonstrated, where every fabrication route provides different capabilities for controlling phase constitution, chemical homogeneity, grain structure, and defect population. While conventional melting and casting remain attractive for bulk material fabrication due to the relative simplicity and scalability, powder metallurgy and mechanical alloying can facilitate refined and nanocrystalline structures. Moreover, additive manufacturing provides further flexibility through rapid solidification and the production of complex geometries. Henceforth, the successful development of HEAs for engineering applications requires an integrated understanding of processing–structure–property relationships [93,315].

The major HEA classes reviewed further illustrate the capabilities of these advanced engineering materials. FCC-based HEAs offer a favorable combination of ductility and damage tolerance, while RHEAs could be applied in severe and intense environments. Nanostructured HEAs can provide enhanced mechanical and functional characteristics through nanoscale structural control, whereas EHEAs offer an attractive combination of strength, ductility, and castability. In addition, HESAs could provide a promising pathway towards high-temperature applications via γ/γ′ microstructures and precipitation-strengthening characteristics. Collectively, these classes have established the versatility of HEA performance through deliberate control of composition and microstructure [316,317].

## Figures and Tables

**Table 1:** Limitations of certain phase parameters and their criteria for application.

Parameter	Criteria	Limitation	Reference
Configurational Entropy (Δ*S*_mix_)	$\Delta S_{\mathrm{mix}}\geq 12\frac{J}{\mathrm{mol K}}$ or 1.5*R*: Solid-phase solution	The threshold of 1.5*R* is of scientific debate, as low configurational entropy will still exhibit HEA properties. Some researchers argue that alloys with less than 5 alloys and the Δ*S*_mix_ ≥ 1.0*R* are also arguably HEAs.	[50,51]
Atomic Size Difference (*δ*)	*δ* ≤ 6.6% (typically, *δ* ≤ 4.5–6.5%): single-phase solid solutions.	It does not identify the solid solution independently.	[59]
Enthalpy of Mixing (Δ*H*_mix_)	$3.2\frac{\mathrm{kJ}}{\mathrm{mol}}\geq \Delta H_{\mathrm{mix}}\geq -11.6\frac{\mathrm{kJ}}{\mathrm{mol}}$: solid phase $\Delta H_{\mathrm{mix}}\leq -12.2\frac{\mathrm{kJ}}{\mathrm{mol}}$: amorphous phase	The parameter rules out intermetallic compounds within the solution. A metastable phase must first be formed to be able to be predicted by the parameter.	[53]
Valence Electron Concentration (VEC)	FCC: VEC ≥ 8.0 FCC + BCC: 6.87 ≤ VEC < 8.0 BCC: VEC < 6.87	VEC parameters do not consider the effect of temperature, as structures may change with temperature. Only valid for 3D or 4D transition metal elements. VEC criteria are mostly summarized as equiatomic materials, whereas non-equiatomic compositions are not yet fully understood. Cannot independently predict phase stability or intermetallic precipitation.	[57]
Solid Solution Parameter (Ω)	Ω ≥ 1.1 and *δ* ≤ 6.6%: single-phase solid solutions	When graphed with the atomic radius, some overlaps between multiphase and single-phase persist, making it impossible to separate the two.	[54]
Single-Phase Solid Solution Parameter (ϕ)	*ϕ* ≥ 1.0 (or system-specific thresholds *ϕ* ≥ 20): stable single-phase formation.	The parameter is unable to identify multiphase or amorphous phase solutions.	[58]

**Table 2:** Effects of adjusting the composition or adding other elements to the structure, and performance of HEAs [46,69,70,71,72,73].

Alloy System	Structural Effect (Phase)	Key Results	Reference
*Fe_x_Cr_y_NiALTi*	A phase transition occurs from FCC to BCC and Heusler-type β′-Ni2TiAl (L21) as the Cr/Fe ratio increases.	The synergistic effects of a soft FCC phase, a hard BCC phase, and a uniform distribution of L21 nanoparticles within the BCC matrix provide the alloy with acceptable strength and excellent tensile properties.	[69]
*Al_x_CoCrFeNi*	Increasing the Al content causes a change from the initial single FCC to an FCC + ordered BCC structure (B2) and then to a duplex BCC structure (A2 + B2).	With an increase in the amount of Al content, it provides a higher hardness property but becomes more ductile.	[70]
*Co_x_CrFeNiMnAl*_0.3_	The stability of the FCC solid solution phase decreases with the reduction in Co content. It also enhances the formation of the BCC phase.	The structure of the BCC phase will gradually become stiffer as the Co content is reduced. Reducing the Co content will also help to limit grain growth of the FCC phase during the recrystallisation process. As a result, the alloy’s tensile strength improved.	[71]
*Al_x_CrFeMnNi* + *Cu_y_*	The low Al content alloy consists of FCC and BCC phases, whereas the high Al content alloys consist of two BCC phases rich in Cr-Fe and Ni–Al.	FCC phase solid solution is stabilized by reducing the Al content and adding up to 3% Cu.	[72]
*CoCrFeMnNi* + *Mo_x_*	The sigma phase presents with the addition of Mo content into the FCC phase.	As the Mo content increased, the yield strength of the alloys increased from 216 to 765 MPa, whereas the strain at break decreased from 50 to 19%, showing its ability to withstand greater mechanical stress before it begins to deform permanently. The optimal compression occurs when the Mo content is added up to 11% to the alloy, which results in a yield strength of 547 MPa, a fracture strength of 2672 MPa, and 44% fracture strain.	[73]

**Table 3:** SFE regimes and their corresponding deformation mechanisms in FCC HEA [83].

SFE Range	Dominant Mechanism
SFE > 40 mJ/m^2^	Conventional dislocation glide
20–40 mJ/m^2^	Mechanical twinning (TWIP)
SFE < 15 mJ/m^2^	Stress-induced martensitic transformation (TRIP)

**Table 4:** Properties of HEA with different Al compositions [239].

Composition	Y.S. (MPa)	UTS (MPa)	Elongation (%)	Elastic Modulus (GPa)	Shear Modulus (GPa)
CAl_0_	240 ± 8	522 ± 17	64	227 ± 4	87 ± 1.5
CAl_2_	253 ± 13	551 ± 13	81	185 ± 3	84 ± 2
CAl_3_	254 ± 12	560 ± 11	82	200 ± 4	82.25 ± 1.35
CAl_4_	268 ± 15	595 ± 8	69	195 ± 3	80.66 ± 1.65
CAl_5_	321 ± 11	718 ± 20	66	199 ± 4	79 ± 2

**Table 5:** Selected RHEAs and their reported mechanical properties at room temperature [254,255,256,257].

RHEA/Material	Mechanical Property at Room Temperature	Key Observation	Significance	Reference
(TiZr)_0.8_(NbTaV)_0.2_	Compressive strength against tension	>50% compressive plasticity; approximately 7% tensile plastic strain	Demonstrates strong tension-compression asymmetry	Z. Liu et al. [254]
AlMo_0.5_NbTa_0.5_TiZr	Fracture toughness	Improved from 4.1 to 4.7 MPa m^1/2^ with increasing cooling rate	Precipitate refinement can improve toughness	P. Suárez Ocaño et al. [255]
Hf_15_Nb_40_Ta_25_Ti_15_Zr_5_	Strength, ductility, and workability	>920 MPa yield strength; >15% elongation with excellent cold workability	Potentially high-temperature structural material	R.R. Eleti et al. [256]
Ti_42.55_V_28.37_Zr_14.19_Nb_14.19_Mo_0.7_	Strength, ductility, and lightweight properties	Approximately 1 GPa yield strength; ~20% uniform degradation; ~5.9 g/cm^3^ density (low)	Good combination of strength, ductility, and low density	Y.F. Jia et al. [257]

**Table 6:** Strengthening mechanisms of RHEAs [77,78,81,258,259,260,261].

Mechanisms	Fundamental Principle	Application(s)	Example(s)	Sources
SSS	Distortion of dislocations due to atomic size and elastic modulus mismatch of principal elements.	Fundamental strengthening mechanism in single-phase BCC RHEAs.	*MoNbTaW* & *MoNbTaVW*	[77,78,258]
GBS	Dislocation transmission restriction due to grain refinement that reduces dislocation pathways.	Controlled solidification, powder metallurgy, hot deformation, recrystallisation, and heat treatment for fine and stable grains.	*H* *f_x_Mo*_0.5_ *NbTa_x_TiV*_1.5−*x*_ *Zr_x_*	[81]
Phase Transformation Strengthening	Combination of TRIP and TWIP to produce a strong and ductile HEA to overcome strength–ductility trade-off.	Applied through metastability engineering for composition adjustment to ensure phase transformation.	*TiZrHf* (*VNbTa*)*_x_*	[259,260]
Precipitation Strengthening	Formation of fine second-phase particles within the structure of HEA to obstruct propagation of dislocation motion.	Controlled thermal treatment to generate stable particles within the matrix of HEAs.	*NbMoTaWVCr*	[261]

**Table 7:** Investigated RHEAs and their high-temperature oxidation behavior [263,264,265,266].

RHEA	Alloying Elements Investigated	Oxidation Conditions	Observed Behavior	Effect on Oxidation Resistance	Reference
ZrTiNbTaSn	Zr and Sn	600–800 °C	Promoted selective oxidation and formation of dense ZrO_2_ and TiO_2_ protective layers.	Improved the oxidation resistance.	[263]
TiZrV_0.5_Nb_0.5_	Al and Si	1000 °C	Al and Si improved the density and adhesion of the oxide film.	Enhanced oxidative protection.	[264]
TiNbTaVW	V	850–1050 °C	Higher temperatures resulted in greater oxidation weight gain, associated with increased V_2_O_5_ volatilization.	Deteriorated oxidation resistance at a higher temperature.	[265]
TaMoZrTiAl	Nb and Si	1000 °C for 20 h	Combined Nb and Si addition contributes to reducing the oxidation weight gain; B1-Nb-Si exhibited the lowest weight gain.	Significantly improved oxidation resistance.	[266]

**Table 8:** Investigated RHEAs and their irradiation resistance.

RHEA/Material	Irradiation Condition	Key Observation	Significance	Reference
NbZrTiV	Proton/He-ion irradiation	Dislocation loops and He bubbles formed, with defect density increasing with irradiation dose.	Demonstrates the response of RHEA to irradiation-induced defects.	H. Wan et al. [272]
VNbMoTaWTi	Proton/He-ion irradiation	Dislocation loops and He bubbles were observed.	Provides insight into irradiation-induced defect evolution in RHEA.	H. Wan et al. [272]
TiVTa	Simulated displacement cascades	Lower point-defect accumulation and clustering than pure V.	Indicates improved resistance to irradiation-induced defects.	Y.P. Zhao et al. [274]
CrMoTaWV	Low-energy, high-flux He-plasma irradiation	Higher threshold for fuzz formation and lower fuzz growth rate than conventional W.	Potential resistance to He-induced surface degradation.	T. Cheng et al. [275]
WTaCrV	Irradiation and annealing	Amorphous-to-crystalline transition occurred.	Indicates that irradiation stability depends on alloy composition and structure.	M.A. Tunes et al. [276]
WTaCrVHf	8 dpa at 1073 K	Maintained amorphous structure; nanoscale Hf precipitates formed.	Enhanced microstructural stability and potential defect-trapping/self-healing behavior.	M.A. Tunes et al. [276]

## Data Availability

No new data were created or analyzed in this study. Data sharing is not applicable to this article.

## Abbreviations

FCC, face-centered cubic; BCC, body-centered cubic; HCP, hexagonal close-packed; SFE, stacking fault energy; TWIP, twinning-induced plasticity; TRIP, transformation-induced plasticity; DCMS, direct current magnetron sputtering; NC, nanocrystalline; MA, mechanical alloying; EHEA, eutectic high-entropy alloy; HESAs, high-entropy superalloys; SSS, solid solution strengthening.
